# Lipid Metabolism‐Driven CNS Repair via Targeted EV Delivery of PAF to Neurons

**DOI:** 10.1002/jev2.70241

**Published:** 2026-02-19

**Authors:** Shih‐Yin Chen, Jing‐Ya Hsu, Chen‐Fu Lo, Yu‐Wei Liu, Wei‐Neng Liao, Wen‐Ting Luo, Yu‐Ju Chen, Jui‐Ping Li, Jen‐Kun Chen, Lun Kelvin Tsou, Hua‐Jung Li

**Affiliations:** ^1^ Institute of Cellular and System Medicine National Health Research Institutes Miaoli Taiwan; ^2^ Institute of Biotechnology National Tsing Hua University Hsinchu Taiwan; ^3^ Institute of Biotechnology and Pharmaceutical Research National Health Research Institutes Miaoli Taiwan; ^4^ Institute of Biomedical Engineering and Nanomedicine National Health Research Institutes Zhunan Miaoli County Taiwan; ^5^ Program in Tissue Engineering and Regenerative Medicine National Chung Hsing University Taichung City Taiwan

**Keywords:** bioorthogonal labelling, mesenchymal stem cells, neural repair, phospholipid, platelet‐activating factor, spatiotemporal delivery, SPECT

## Abstract

Platelet‐activating factor (PAF) is a potent phospholipid mediator with therapeutic potential in neuroregeneration, but its therapeutic application is hindered by rapid degradation and systemic proinflammatory effects. Here, we present an engineered extracellular vesicle (EV)‐based delivery strategy that stabilizes and targets PAF to sites of hippocampal injury, restoring neuronal structure and cognitive function. EVs derived from EP4 antagonist‐primed mesenchymal stem cells (GWEVs) exhibit enhanced secretion and selective enrichment of bioactive lipids, particularly PAF, which promotes neuroregeneration, attenuates gliosis and rescues spatial memory. Mechanistic studies reveal that PAF's therapeutic activity depends not on classical PTAFR engagement but on neuronal metabolism via PAF‐acetylhydrolase (PAFAH), particularly the PAFAH1B1 subunit. The hydrolysis‐resistant analogue MPAF fails to confer benefit, underscoring the requirement for enzymatic processing. To address translational needs, we developed a bioorthogonal click‐labelling platform that enables real‐time SPECT imaging of EV biodistribution while preserving function. GWEVs preferentially accumulate in injured hippocampi, confirming targeted delivery. This study defines a previously unrecognized lipid metabolism‐dependent repair mechanism and demonstrates the feasibility of leveraging EVs for CNS‐targeted delivery of labile lipid therapeutics. These findings offer a platform for advancing regenerative strategies in neurodegenerative diseases and traumatic brain injury.

## Graphical abstract

Encapsulation within GWEVs stabilizes platelet‐activating factor (PAF), prevents peripheral inflammation and enables targeted delivery across the blood–brain barrier. In the brain, GWEVs localize to injury sites and deliver PAF to neurons, where PAFAH1B1‐dependent cytoskeletal remodelling promotes repair, supporting GWEVs as a therapeutic platform for neural regeneration.

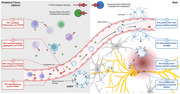

## 1 Introduction

Extracellular vesicles (EVs) derived from mesenchymal stem cells (MSCs) have emerged as a promising acellular tool for regenerative medicine (Bhat et al. 2024). These nanoscale vesicles transport a diverse cargo of proteins, lipids and nucleic acids, enabling them to modulate key biological processes including immune responses, tissue repair and intercellular communication. As cell‐free vectors of bioactive molecules, MSC‐EVs offer advantages over cell‐based therapies, such as reduced immunogenicity and lower tumorigenic risk. However, despite their therapeutic potential, clinical translation is largely hindered by critical challenges, including low yield, heterogeneous cargo composition (Almeria et al. 2022), inefficient tissue targeting and a lack of well‐defined efficacy markers (Bhat et al. 2024, Gimona et al. 2021).

Recent studies have shown that priming MSCs with diverse stimuli such as inflammatory cytokines (Ragni et al. 2020), hypoxia (Lo Sicco et al. 2017), oxidative stress (Phinney et al. 2015), pharmacological agents (Mendt et al. 2018, Chen et al. 2020, Chen et al. 2019) and physical inputs (Kusuma et al. 2022), can enhance EV biogenesis, cargo enrichment and therapeutic potency by influencing the MSC functional phenotype and modulating their secretome. Such priming strategies enable fidelity in EV function and serve as a standard for the development of high‐efficacy EV‐based therapeutics with quality controls. Among these, inhibition of prostaglandin E2 (PGE_2_) signalling—particularly via the EP4 receptor (Chen et al. 2019, 2020)—has emerged as a promising but underexplored regenerative approach, supported by emerging evidence suggesting PGE_2_/EP4 signalling as a regenerative target (Lin et al. 2017, Wang et al. 2025). PGE_2_ is a central mediator of inflammation (Kalinski 2012, Sugimoto and Narumiya 2007) and stem cell behaviour (Vasandan et al. 2016, Hoggatt et al. 2009). Modulation of EP4 pathway presents a novel mechanism for altering the immunomodulatory cargo of EVs; however, this avenue requires deeper mechanistic studies. Nonetheless, with the marked increase in EV production, EP4 modulation also provided a level of control over their functional cargo (Chen et al. 2019, 2020), addressing a critical bottleneck in the standardization and scalability of EV‐based therapeutics.

In this study, we examined the effect of selective EP4 receptor inhibition on MSCs using the EP4 receptor antagonist GW627368X (Chen et al. 2019, 2020), focusing on its ability to modulate EV biogenesis and metabolic content. We found that EP4 receptor blockade not only increased EV yield but also enriched vesicles with bioactive molecules, including the potent signalling lipid, platelet‐activating factor (PAF), which plays roles in both immune regulation and neural development. Acting via its G protein‐coupled receptor PTAFR, PAF promotes inflammation by recruiting neutrophils and eosinophils, increasing cytokine secretion, for example, IL‐6, IL‐1β and TNF‐α, and enhancing vascular permeability (Upton et al. 2022). PAF also supports tissue remodelling in wound healing and ischemia by promoting angiogenesis and endothelial cell proliferation (Bhosle et al. 2016, Montrucchio et al. 2000), and plays key roles in the central nervous system, regulating synaptogenesis, neurotransmitter release and adult neurogenesis (Dalmaso et al. 2024, Bazan and Allan 1996). Importantly, defects in ether lipid metabolism, including the PAF hydrolysis pathway, have been linked to severe neurodevelopmental disorders (Dorninger et al. 2017). Together, these insights underscore the dual roles of PAF as both an immune modulator and a neurodevelopmental signalling molecule.

The neuro‐repairing ability of PAF is significantly undermined by its instability in peripheral circulation and the proinflammatory activation associated with PTAFR. To circumvent these challenges, we demonstrated that EVs derived from EP4 antagonist‐primed MSCs, referred to as GWEVs, can serve as a stable and targeted delivery system for PAF, enhancing its biodistribution for neural repair. In a mouse model where hippocampal CA1 pyramidal neurons are selectively ablated using doxycycline‐regulated diphtheria toxin A (DTA) expression, our findings demonstrated that PAF‐enriched GWEVs effectively restore neuronal structure and cognitive function, as evidenced by histological, molecular and behavioural assessments. To elucidate the mechanism of action, we further showed that the bioactivity of PAF is contingent upon enzymatic processing by PAF‐acetylhydrolase (PAFAH), employing a hydrolysis‐resistant analogue, methylcarbamyl PAF (MPAF), as a control. To confirm the spatiotemporal accumulation of GWEVs, we utilized a bioorthogonal click‐chemistry‐based labelling strategy to track their in vivo distribution. Traditional lipid dyes often compromise the functionality of EVs; however, our fluorescence or radioisotope click‐labelled GWEVs maintain the bioactivity of sensitive lipid mediators like PAF, effectively promoting neuroregeneration through a PAFAH‐dependent mechanism while preferentially accumulating in injured brain regions. These results underscore the importance of biodistribution in therapeutic efficacy, highlighting GWEVs as a promising delivery platform for lipid‐based therapeutics in the injured central nervous system (CNS). This innovative approach not only harnesses the regenerative effects of PAF but also minimizes systemic exposure and receptor‐mediated side effects, paving the way for enhanced therapeutic strategies in neuro‐repair.

## 2 Materials and Methods

### 2.1 Cell Culture

The different batches of human bone marrow MSCs (passage first) were obtained from Sciencells (Carlsbad, CA). MSCs were propagated in low glucose Dulbecco's modified Eagle's medium containing 10% hPL. EVs in hPL were removed by ultracentrifugation before hPL was used for MSC culture (Thery et al. 2006). MSC at the fifth passage were used for EV collection. MSCs were discarded after one round of EV collection. NE‐4C cells, neuroectodermal stem cells established from the cerebral vesicles of 9‐day‐old mouse embryos lacking the functional p53, were obtained from the American Type Culture Collection. NE‐4C cells were propagated in Eagle's Minimum Essential Medium containing 10% foetal bovine serum with penicillin‐streptomycin.

### 2.2 Primary Hippocampal and Cortical Neuron Culture

Timed‐pregnant C57BL/6 mice at embryonic day 17.5 (E17.5) were euthanized via CO_2_ inhalation in accordance with institutional animal care protocols. Embryos were excised from the uterus, placentas removed and placed into ice‐cold CMF‐HBSS, calcium‐ and magnesium‐free HBSS (Thermo Fisher, 14185) containing 1 mM HEPES (Thermo Fisher, 15630) and 100 unit/mL Penicillin‐streptomycin (Thermo Fisher, 15140). Brains were dissected under a stereomicroscope in a sterile laminar flow hood. Each embryo was stabilized by the neck using fine forceps, and the cranial skin and skull were gently incised along the midline. The cerebral cortices were isolated from both hemispheres, and hippocampi were carefully dissected from the medial cortical wall. Isolated cortices and hippocampi were transferred separately into tubes containing 5 mL of pre‐warmed 0.25% Trypsin/DETA (Thermo Fisher, 25200) with 1 mM HEPES and incubated at 37°C for 25 min. Post‐digestion, tissues were rinsed twice with pre‐warmed CMF‐HBSS for 3 min per wash to eliminate residual enzymes. Samples were resuspended in CMF‐HBSS containing 0.3 mg/mL DNase I (Merck, DN25) and subjected to mechanical trituration, first with a 10 mL serological pipette (10 strokes) and then with a pipette fitted with a 1000 µL tip (10 strokes). The resulting cell suspension was centrifuged at 100 × *g* for 10 min at room temperature. Supernatants were discarded, and cell pellets were gently flicked to disperse, then resuspended in pre‐warmed neurobasal medium (Thermo Fisher, 21103) supplemented with 1X B‐27 (Thermo Fisher, 17504), and 0.5 mM L‐glutamine (Merck, G8540). Viable cells were quantified using trypan blue exclusion with a hemocytometer. To prepare neuron‐conditioned medium, primary cortical neurons (2.3 × 10^6^ cells per 10 cm dish) were cultured on PLL‐coated dishes in 15 mL of neuronal maintenance medium. After 7 days in vitro, the medium was harvested, filtered and stored at −20°C for subsequent use. For hippocampal neuron culture, 10,000 viable cells per well were plated onto 12 mm glass coverslips pre‐coated with 450 µL of 100 µg/mL poly‐L‐lysine (PLL; Merck, P2636) in 24‐well plates, using 500 µL of neuron‐conditioned medium per well. After a 2‐h adhesion period at 37°C and 5% CO_2_. Treatments were applied by mixing EVs or compounds, as specified in figure legends, with 1× PBS and adding 50 µL per well. Neurons were maintained for 3 days under standard incubator conditions (37°C, 5% CO_2_) before fixation and downstream analysis.

### 2.3 Immunofluorescence

Neurons were fixed with 3.7% formaldehyde (v/v) in 1× PBS pre‐warmed to 37°C for 15 min. After fixation, cells were washed three times with 1× PBS to remove residual fixative. Fixed cells were permeabilized using 0.25% Triton X‐100 in PBS for 5 min at room temperature, followed by three washes with PBS. Non‐specific antibody binding was blocked by incubation with 10% bovine serum albumin (BSA) in PBS for 30 min at 37°C. After removing the blocking buffer, cells were incubated for 1 h at 37°C with anti‐β3 tubulin primary antibodies (BioLegend, 801202; 1:3000 dilution), anti‐SOX2 (Santa Cruz Biotechnology, sc‐17 320; 1:250 dilution), anti‐NeuN (Merck Millipore, ABN78; 1:250 dilution, or anti‐Nestin (BioLegend, 839801; 1:250 dilution) diluted in PBS. Following three washes with PBS, secondary antibodies (Alexa Fluor 488‐conjugated anti‐mouse IgG, 1:1000 in PBS with 2% BSA) were applied along with DAPI (10 µg/mL) for nuclear staining. Coverslips were washed and mounted for imaging. Total neurite length was quantified using NeuronJ, and neurite complexity was assessed via Sholl analysis.

### 2.4 EV Isolation

EVs were isolated from MSC culture media by differential ultracentrifugation as previously described (Chen et al. 2019, 2020). Briefly, MSCs were treated with dimethyl sulfoxide (DMSO) vehicle or 20 µg/mL EP4 antagonist GW627368X (GW) for 6 days. Culture media were collected and were replaced with fresh media supplemented with DMSO or GW every 3 days. The collected culture media were centrifuged at 300 × *g* for 5 min to remove cells (P1), at 1200 *g* for 20 min (P2), then at 10,000 × *g* for 30 min (P3) all at 4°C. Finally, EVs (P4) were separated from the supernatant by centrifugation at 110,000 × *g* for 70 min. The EV pellet was washed once in phosphate‐buffered saline (PBS) and then resuspended in PBS for further analysis and injection. EV number was quantified by Nano‐flow cytometric analysis, and total protein content was measured using the BCA assay.

### 2.5 Electroporation of EVs

EVs (2.5 × 10^10^ EVs in 240 µL of PBS) were mixed with 10 µg of PAF antibody (Bioss, BS‐1119R; 1 µg/µL) or 10 µg of control IgG (Cell Signalling, 2729; 1 µg/µL) in 250 µL of 2X electroporation buffer (0.05M KCl, 0.0023M KH_2_PO_4_, pH 7.2). Electroporation was performed in a 4 mm cuvette using a Gene Pulser Xcell Electroporation System (Bio‐Rad), as previously described (Alvarez‐Erviti et al. 2011; Melo et al. 2014). Three independent electroporation reactions were carried out and pooled for purification using centrifugal filters, followed by an additional wash with 2.5 mL PBS to remove unincorporated antibodies. The purified EVs were then resuspended in PBS for further analysis. EV concentration was quantified by nano‐flow cytometric analysis prior to downstream applications.

### 2.6 Animal Experiments

All research involving animals complied with protocols approved by the NHRI Committee on Animal Care. B6.CBA‐Tg(Camk2a‐tTA) and B6.Cg‐Tg(tetO‐diphtheria toxin A [DTA]) mice were obtained from Jackson Lab. Doxycycline (Dox) was removed from the diet of 6‐week‐old tetO‐DTA mice and Camk2a‐tTA/tetO‐DTA mice for 30 days. On the 31st day, doxycycline (2000 ppm) was returned to the mouse chow. Mice were maintained on tetracyclineenriched chow, except for the 30‐day Dox‐free period for brain lesion. After the Dox‐free period, mice were injected with 100 µL PBS, PAF in PBS (1.5 µg/kg body weight/injection, twice), MPAF in PBS (1.5 µg/kg body weight/injection) or GWEVs, via intracardiac or intravenous injection as indicated in the figure legends. After the injection, mice were subjected for behavioural analysis (e.g., novel object recognition test [NORT], novel location recognition test [NLRT], Morris water maze [MWM]) at the time points indicated in the figure legends. Mice were sacrificed at the time points indicated in the figure legends and the brains were collected for further analysis including H&E staining and immunolabelling. Single‐transgenic tetO‐DTA littermates maintained on Dox served as undamaged controls (UC).

### 2.7 Animal Behaviour Examination

All cognition, learning and memory tests were performed as described previously (Chen et al. 2019, 2020). The numbers of animals for each behavioural group are indicated in the figure legends. Each mouse received only one behavioural test per day. In NORT and NLRT, each mouse was allowed to explore the objects for 5 min (exploratory phase) and then was returned to the cage for another 5 min. After the 5‐min interval in the cage, the mouse was returned to (a) the chamber with the previously exposed object and a novel object (NORT) or (b) the chamber in which one of the two objects was displaced from its original position (NLRT), for a 3‐min test phase. Exploration counted as positive if the mouse's head was within one inch of the object with neck extended and vibrissae moving. The exploratory phase and test phase were videotaped to measure (time for exploring novel object or location)/(time for total exploring).

In the MWM test, the learning trials were performed at the same time on Day 1 to Day 4. The trial began from a different quadrant of the pool for each day (second and third quadrant for Day 2, third and fourth quadrant for Day 3, first and fourth quadrant for Day 4). Each trial ended when the mouse arrived at the platform, or after 60 s had passed. Mice were immediately removed from the pool at the end of the trial. All tracks from all trials were recorded and analysed using the Videotrack software (Viewpoint).

### 2.8 Tissue Preparation and Immunofluorescence From Tissue Sections

Paraformaldehyde‐fixed tissues were embedded in paraffin blocks and cut into 4‐µm sections. Haematoxylin and eosin staining was conducted according to conventional procedures. Tissue sections were deparaffinized/hydrated and were then subjected to antigen retrieval in citrate buffer (pH 6.0) for 10 min. The sections were incubated with primary antibodies overnight at 4°C and then with secondary antibodies for 1 h at room temperature. Cell nuclei were visualized with DAPI. Slides were mounted with ProLong Gold Antifade Reagent and imaged using a TCS SP5 II confocal microscope. The following antibodies were used: anti‐GFAP (Merck Millipore, Mab360; 1:250 dilution), anti‐Iba1 (Abcam, ab5076; 1:250 dilution), anti‐β3 tubulin (Cell Signalling, 5568; 1:250 dilution), anti‐SOX2 (Santa Cruz Biotechnology, sc‐17 320; 1:250 dilution), anti‐MAP2 (Merck Millipore, AB5622; 1:250 dilution), anti‐NeuN (Merck Millipore, ABN78; 1:100 dilution) and anti‐DCX (Santa Cruz Biotechnology, sc‐8066; 1:250 dilution). Signal quantification was performed using ImageJ.

Immunohistochemistry quantification was performed using ImageJ, following the ImageJ User Guide. For quantitation of glial fibrillary acidic protein (GFAP)‐positive cells, SOX2‐positive cells, NeuN‐positive cells and Iba1‐positive cells, 8‐bit images of the hippocampus CA1 region were loaded into ImageJ. The signal‐positive area was calculated using “Analyze Particles” under the ImageJ “Analyze” function. For quantitation of microtubule associated protein 2 (MAP2)‐positive areas and β3 tubulin‐positive areas, 8‐bit images covering of the hippocampus CA1region were loaded into ImageJ. The signal‐positive area was calculated using “Analyze Particles” under the ImageJ “Analyze” function. For quantification of CA1 neuron thickness, 8‐bit DAPI images covering whole hippocampi were loaded into ImageJ. For each hippocampus, three lines across the DAPI‐positive layer of neuronal nuclei in CA1 region were drawn and lengths of the lines were measured using ImageJ. The average length of the three lines represents the CA1 neuron thickness of the hippocampus.

### 2.9 Quantification of CA1 Pyramidal Neurons With QuPath

H&E‐stained coronal mouse brain sections were scanned at 40× magnification using a digital slide scanner. High‐resolution whole‐slide images were exported as SVS files and analysed in QuPath (Bankhead et al. 2017), using the built‐in Object Classifier tool for cell detection. The CA1 region of the hippocampus was manually annotated using QuPath's polygon tool, guided by a standardized mouse brain atlas. To distinguish pyramidal neurons from other cell types and background structures, QuPath's object classifier was trained using supervised learning. Initially, the Cell Detection tool was used to identify all nuclei based on haematoxylin optical density. A minimum of 50 pyramidal neurons and 50 non‐neuronal cells were manually labelled using the “Annotations” panel. The classifier was trained on cellular features including nuclear area, circularity, staining intensity and texture to optimize classification accuracy. Once trained, the model was applied to batch process all annotated hippocampal regions of interest (ROIs). Pyramidal neurons were visualized in yellow and non‐neuronal cells in blue. Cell counts were normalized by area for each hippocampal ROI.

### 2.10 Western Blotting

Total protein of cells or EVs was extracted with RIPA lysis buffer. The same amount EV protein or EV protein samples from the same number of EVs were loaded in each lane. Protein lysates were resolved on a 10%–12% Bis‐Tris Gel, transferred to PVDF membranes, probed with primary antibodies (1:1000 dilution) overnight at 4°C and then with HRP‐linked secondary antibodies (1:3000 dilution) and visualized with ECL reagent. The following antibodies were used: anti‐GAPDH (GeneTex, GTX100118), anti‐CD44 (R&D system, BBA10), anti‐CD24 (Merck, SAB1402713), anti‐CD63 (GeneTex, GTX132953), anti‐HSP70 (GeneTex, GTX111088), anti‐CD81 (GeneTex, GTX101766), anti‐CNP (GeneTex, GTX103954) and anti‐β3 tubulin (Cell Signalling Technology, CS5568).

### 2.11 Immunoprecipitation

Cells were lysed in ice‐cold PBS containing 0.5% IGEPAL CA‐630/Nonidet P‐40 (Merck, I8896), 5% bovine serum albumin (BSA) and protease inhibitor cocktail for 40 min at 4°C with gentle rocking. Lysates were clarified by centrifugation at 16,000×*g* for 10 min at 4°C. Protein concentration in the resulting supernatant was quantified using the BCA assay, and 500 µg of total protein in 1 mL was used for each immunoprecipitation reaction. The lysate was pre‐cleared with 20 µL of Protein A‐Sepharose beads (Thermo Fisher Scientific, 20421) for 1 h at 4°C under constant rotation to reduce nonspecific binding. After centrifugation, the supernatant was transferred to fresh tubes and incubated overnight at 4°C with diluted primary antibodies specific for the target proteins: 20 µg/mL anti‐PAFAH1B1 (Merck Millipore, L7391) or 2 µg/mL anti‐DCX (Santa Cruz Biotechnology, sc‐806620). Following overnight incubation with antibodies, 20 µL of Protein A/G Agarose beads were added to each tube and further incubated for 3 h at 4°C with gentle mixing to capture the immune complexes. The bead‐bound complexes were washed six times with PBS containing 0.5% IGEPAL CA‐630/NP‐40 to eliminate non‐specifically bound proteins. Protein complexes were eluted by boiling the beads in 2× Laemmli SDS‐PAGE sample buffer for 5 min. The precipitated proteins were resolved by 12% SDS–PAGE and were analysed with western blotting.

### 2.12 Metabolomic Profiling

Metabolomic analysis of EVs was conducted to comprehensively characterize their small‐molecule content. EVs isolated from MSC cultures—pooled pellets obtained from ten 15‐cm culture dishes—were subjected to biphasic extraction using a modified methanol–chloroform–water protocol optimized for partitioning both polar and non‐polar metabolites. The extraction was initiated by resuspending each EV pellet in 1 mL of ice‐cold water and 3.75 mL of a pre‐mixed chloroform:methanol solution (1:1, v/v). The suspension was vortexed vigorously for 10 min to ensure thorough disruption of vesicle membranes and solubilization of metabolite contents. Subsequently, 1.25 mL of chloroform was added, followed by 1 min of vortexing, and then 1.25 mL of water was introduced with another 1‐min vortex. The mixture was centrifuged at 13,000 × *g* for 10 min at 4°C to facilitate partitioning into aqueous (upper) and organic (lower) phases. Both layers were carefully collected into separate tubes and dried using vacuum centrifugation. Dried extracts were reconstituted in 50 µL of a chloroform:methanol (2:1, v/v) mixture immediately prior to analysis. Metabolite separation and identification were performed using liquid chromatography‐tandem mass spectrometry (LC‐MS/MS), employing both positive and negative electrospray ionization (ESI) modes. Operating in dual polarity mode significantly enhanced metabolome coverage, enabling detection of a broad spectrum of charged species.

### 2.13 Chemistry

Reactions requiring anhydrous conditions were performed in flame‐dried glassware and cooled under an argon or nitrogen atmosphere. Unless otherwise stated, reactions were carried out under argon or nitrogen and monitored by analytical thin layer chromatography performed on glass‐backed plates (5 × 10 cm) precoated with silica gel 60 F254 as supplied by Merck (Merck & Co., Inc., Whitehouse Station in Readington Township, NJ, USA). The resulting chromatograms were visualized by looking under an ultraviolet lamp (λ = 254 nm). Solvents for reactions were dried and distilled under an argon or nitrogen atmosphere prior to use as follows: THF, diethyl ether (ether) and DMF from a dark blue solution of sodium benzophenone ketyl; toluene, dichloromethane and pyridine from calcium hydride. Flash chromatography was used routinely to purify and separate product mixtures using silica gel 60 of 230−400 mesh size as supplied by Merck. Eluent systems are given in volume/volume concentrations. 1H spectra were recorded on a Bruker Ascend 400 spectrometer. Chemical shift values are reported in ppm relative to the TMS in delta (δ) units. Multiplicities are recorded as s (singlet), br s (broad singlet), d (doublet), t (triplet), q (quartet), dd (doublet of doublets), dt (doublet of triplets) and m (multiplet). Coupling constants (J) are expressed in hertz. Electrospray mass spectra (ESMS) were recorded as m/z values using an Agilent 1100 MSD mass spectrometer. All test compounds displayed more than 95% purity as determined by Agilent 1100 series HPLC system using a C18 column (Thermo Golden, 4.6 mm × 250 mm). The gradient system for HPLC separation was composed of MeOH (mobile phase A) and H2O solution containing 0.1% trifluoro‐acetic acid (mobile phase B). IUPAC nomenclature of compounds was determined with ACD/Name Pro software.

### 2.14 Synthesis of ((1R,8S,9r)‐bicyclo[6.1.0]non‐4‐yn‐9‐yl)methyl (4‐nitrophenyl) carbonate, BCN‐NC

To a solution of 1,5‐cyclooctadiene (1.06 kg, 9.82 mol, 1.21 L, 8.00 eq.) and Rh2(OAc)4 (2.71 g, 6.13 mmol., 0.005 eq.) in DCM (560 mL) was added a solution of SM3 (ethyl diazoacetate) (140 g, 1.23 mol., 1.00 eq.) in DCM (280 mL) dropwise at 20°C over a period of 3 h under N2. The mixture was stirred at 20°C for 12 h. SM3 was consumed completely and a new spot was detected on TLC (petroleum ether/EtOAc = 50/1, product Rf = 0.25). The combined organic layer was concentrated in vacuum to give a crude product. The crude product was purified by flash chromatography on a silica gel eluted with petroleum ether/EtOAc (1/0 to 50/1). Compound 5 (75.0 g, 386 mmol., 31% yield; Figure S7A) was obtained as a yellow oil.

To a solution of LiAlH4 (30.2 g, 797 mmol., 1.00 eq.) in THF (620 mL), cooled the mixture to 0°C, was added a solution of compound 5 (155 g, 797 mmol., 1.00 eq.) in THF (155 mL) dropwise at 0°C. The mixture was stirred at 25°C for 2 h. compound 5 spot was consumed completely and a new spot was appeared on TLC. The reaction mixture was quenched by Na2SO4.10H2O (250 g) at 0°C and filtered. The combined organic layer was concentrated in vacuum to give a crude product. The crude product was purified by flash chromatography on a silica gel eluted with petroleum ether/EtOAc (from 100/1 to 3/1). Compound 6 (120 g, 788 mmol., 99% yield; Figure S7A) was obtained as a yellow oil (RF 0.20, petroleum ether/ EtOAc = 2/1).

To a solution of compound 6 (110 g, 722 mmol., 1.00 eq.) in DCM (330 mL) was added a solution of Br2 (127 g, 794 mmol., 40.9 mL, 1.10 eq.) in DCM (330 mL) at 0°C under N2. Then the mixture was stirred at 0°C for 0.5 h. Compound 6 spot was consumed completely and a new spot was detected on TLC. The reaction mixture was added Na2SO3 (1.00 L) and extracted with DCM (300 mL, 200 mL × 3). The combined organic phases were washed with brine (100 mL) and dried over Na2SO4. The mixture was filtered and the filtrate was concentrated in vacuum to give a crude product. The crude product was purified by flash chromatography on a silica gel eluted with petroleum ether/EtOAc (from 100/1 to 5/1). Compound 7 (179 g, 573 mmol., 79% yield; Figure S7A) was obtained as a yellow oil (petroleum ether/EtOAc = 3/1, product Rf = 0.35).

To a solution of compound 7 (163 g, 523 mmol., 1.00 eq.) in THF (980 mL) was added t‐BuOK (1 M, 2.10 L, 4.00 eq.) at 0°C. Then the mixture was stirred at 80°C for 12 h. Compound 7 was not consumed completely and two new spots were detected on TLC. The reaction mixture was poured into NH4Cl (800 mL) with stirring, and then extracted with EtOAc (300 mL, 200 mL × 3). The combined organic phases were washed with brine (150 mL) and dried over Na2SO4. The mixture was filtered and the filtrate was concentrated in vacuum to give a crude product. The crude product was purified by flash chromatography on a silica gel eluted with petroleum ether/EtOAc (from 100/1 to 5/1). Compound 8 (34.0 g, 226 mmol., 43% yield; Figure S7A) was obtained as a yellow oil (RF 0.30, petroleum ether/EtOAc = 3/1).

To a solution of compound 8 (24.5 g, 163 mmol., 1.00 eq.) in DCM (85.0 mL) cooled to 0°C was added pyridine (32.2 g, 407 mmol., 32.9 mL, 2.50 eq.) and (4‐nitrophenyl) carbonochloridate (37.8 g, 187 mmol., 1.15 eq.). The mixture was stirred at 25°C for 2 h. Compound 8 was consumed completely and a new spot was detected on TLC. The reaction mixture was concentrated under vacuum to give a crude product. The crude product was purified by flash chromatography on a silica gel eluted with petroleum ether/EtOAc (from 100/1 to 5/1). Compound 9 (31.0 g, 93.9 mmol., 60% yield; Figure S7A) was obtained as a white solid (petroleum ether/EtOAc = 4/1, product Rf = 0.55).

### 2.15 Synthesis of Azido Rhodamine

Rhodamine B (SM1, 1 g, 2.31 mmol) was added, respectively, to 1‐hydroxybenzotriazole hydrate (HOBt, 500 mg, 3.70 mmol), [1‐ethyl‐3(3‐dimethylpropylamine) carbodiimide] (EDCI, 500 mg, 3.22 mmol) and N‐Boc‐piperazine (500 mg, 2.68 mmol). The mixture was dissolved in 200 mL of dichloromethane (DCM) and stirred at room temperature for 4 h. 200 mL of sodium bicarbonate solution was added for extraction. The organic layer (DCM) was treated with Na_2_SO_4_(s) for drying. Finally, the solution was concentrated under reduced pressure to obtain compound 1 (Figure S7B). Without further purification, compound 1 was dissolved in 200 mL of methanol. Then, 3 mL of 6 M HCl aqueous solution was added, and the mixture was heated to reflux for 1 h. Vacuum concentration was performed to remove methanol from the mixture. The resulting compound was then dissolved in 200 mL of DCM. Next, 200 mL of sodium bicarbonate solution was added for workup, and the organic layer was subsequently treated with Na_2_SO_4_(s) to dry the organic fraction. Finally, vacuum concentration was performed again to dry the mixture to yield 1 g of compound 2 (1.81 mmol; Figure S7B). A mixture of crude compound 2 (100 mg, 0.19 mmol), 6‐azido‐hexanoic acid (50 mg, 0.31 mmol), EDCI (50 mg, 0.32 mmol) and HOBt (50 mg, 0.37 mmol) in 10 mL of DCM at room temperature for 15 h. Sodium bicarbonate solution (10 mL) was added for extraction. The organic layer (DCM) was treated with Na_2_SO_4_(s) for dehydration. Finally, the solution was concentrated under reduced pressure to obtain compound 3 (Figure S7B). Column chromatography was used to purify compound 3, with silica gel as the stationary phase. Impurities were washed through the column using the initial mobile phase (10% MeOH/DCM). The ratio of methanol in the mobile phase was gradually increased to 15%, eluting compound 3 based on its polarity and interaction with silica gel. Solvents were removed from the collected fractions under vacuum, yielding 60 mg of compound 3, azido rhodamine (0.09 mmol, 47%).

### 2.16 Synthesis of Azido DOTA

DOTA‐NHS‐ester (SM2, 60 mg, 0.12 mmol) was dissolved in DMF with 2‐{2‐[2‐(2‐Azido‐ethoxy)‐ethoxy]‐ethoxy}‐ethylamine (60 mg, 0.27 mmol), and DIPEA (50 mg, 0.38 mmol) was added. The mixture was stirred at room temperature for 15 h. The mixture was dropped into cold ether. The mixture was then placed in a refrigerator at −30°C for 1 h to allow complete precipitation. The mixture was centrifuged to separate the precipitate from the supernatant. The precipitate was dissolved in distilled water and through gentle stirring or mild heating if necessary. The solution was transferred to a freeze dryer and lyophilized completely under vacuum. After freeze‐drying, the resulting solid product weighing 68 mg (compound 4, azido DOTA, 0.11 mmol, 91%; Figure S7C) was collected.

### 2.17 EV Flow Cytometry

For Di‐8‐ANEPPS membrane staining, 2 µg EVs were resuspended in 100 µL of PBS containing 0.1% Pluronic F‐127 (Thermo Fisher, P6866) and 0.25 µg/mL Di‐8‐ANEPPS (Cayman Chemical, 19541). The suspension was incubated for 1 h at room temperature in the dark to allow incorporation of the membrane potential‐sensitive dye. After incubation, EVs were either directly analysed or subjected to further processing as indicated below. For conjugation via strain‐promoted azide–alkyne cycloaddition (SPAAC), 2 µg of EVs were resuspended in 100 µL PBS containing BCN‐NC and incubated for 1 h at room temperature. The reaction mixture was then diluted 17.5‐fold with PBS and ultracentrifuged at 110,000 × *g* for 70 min at 4°C to remove unbound BCN‐NC. The EV pellet was resuspended in 250 µL PBS containing azido rhodamine and incubated for 30 min at room temperature in the dark. Following labelling, EVs were diluted 30‐fold in PBS and analysed using a CytoFLEX Nano Flow Cytometer (Beckman Coulter) operated in small particle detection mode. Samples were acquired at a flow rate of 1 µL/min for 1 min. Gating strategies were applied to exclude background and instrument noise using nanoViS nanoscale sizing standards (Beckman, D03231) and dye‐only controls. The VSSC1 threshold for defining the EV population was determined individually for each experiment by comparing PBS‐only controls, detergent‐treated EV samples, and untreated EV samples, and was set at the scatter value yielding the largest difference in particle counts between untreated and detergent‐treated EVs. Data were analysed using *FlowJo* software. Details of the nanoscale flow‐cytometry experiments are reported in accordance with the MIFlowCyt‐EV framework and provided in Table S1.

### 2.18 Radiolabelling and SPECT Imaging

To facilitate noninvasive in vivo tracking, EVs, GWEVs, were radiolabelled with gallium‐67 (^67^Ga, half‐life: 78.3 h) via the bioorthogonal click chemistry approach. Radiolabelling was accomplished by first labelling the vesicles with BCN‐NC to introduce strained alkyne groups on surface amines. Separately, ^67^Ga was chelated to azido‐DOTA to enable radiochemical conjugation via strain‐promoted azide–alkyne cycloaddition (SPAAC). The ^67^Ga‐DOTA complex was synthesized by incubating ^67^GaCl_3_ with azido‐DOTA at pH 5.0 in sodium acetate buffer at 90°C for 30 min. Radiolabelling yields were 50%–65% and then purified by Sep Pak C18 cartridge. Radiochemical purities were verified by instant thin‐layer chromatography (ITLC), setting >80% as an acceptable quality control criterion. The ^67^Ga‐DOTA was then reacted with GWEV‐BCN at room temperature for 1 h with gentle agitation. Radiolabelled GWEVs (^67^Ga‐GWEVs, 50–100 µCi/mouse) were administered via intravenous or intracardiac injection under isoflurane anaesthesia. Whole‐body single‐photon emission computed tomography (SPECT) images were acquired using a high‐resolution preclinical SPECT/CT (nanoScan SPECT/CT, Mediso Medical Imaging Systems, Budapest, Hungary) at 4, 24 and 48 h post‐injection. Imaging co‐registration with CT allowed anatomical localization of radiolabelled vesicles. After the final imaging time point, animals were euthanized and major organs—including brain, lungs and heart—were harvested. Biodistribution of ^67^Ga‐GWEVs was quantified by an automatic gamma counter (2480 WIZARD^2^, PerkinElmer, Turku, Finland), and brain tissues were freshly dissected for autoradiography to visualize tissue‐level EV distribution.

### 2.19 PKH26 labelling of EV and Systemic Administration

EVs were labelled using 2 µM PKH26 fluorescent cell linker dye. After incubation for 5 min at 4°C, the EVs were washed twice with PBS and centrifuged at 110,000 × *g* for 60 min. A parallel sample containing only PKH26 and PBS, processed in the same manner, was used as the negative control for the systemic administration. Labelled EVs and the negative control were administered into mice by intracardiac injection or tail vein injection. At 16 h after the injection, mice were sacrificed and the brains were collected for further analysis. Six micrometer cryosectins of mouse brains were stained with DAPI. Slides were mounted with ProLong Gold Antifade Reagent and imaged using a TCS SP5 II confocal microscope.

### 2.20 Statistical Analysis

Data are presented as mean ± SEM. Statistical comparisons between two groups were performed using unpaired *t*‐tests, unless otherwise indicated. For comparisons involving more than two groups, one‐way ANOVA followed by Tukey's multiple comparisons test was used (Figures 5, 6 and S6D). All statistical analyses were performed using GraphPad Prism 9. A *p* value ≤ 0.05 was considered statistically significant.

## 3 Results

### 3.1 EP4 Antagonist Enhances MSC‐Derived EV Secretion and Selectively Enriches PAF Content

**FIGURE 1:**
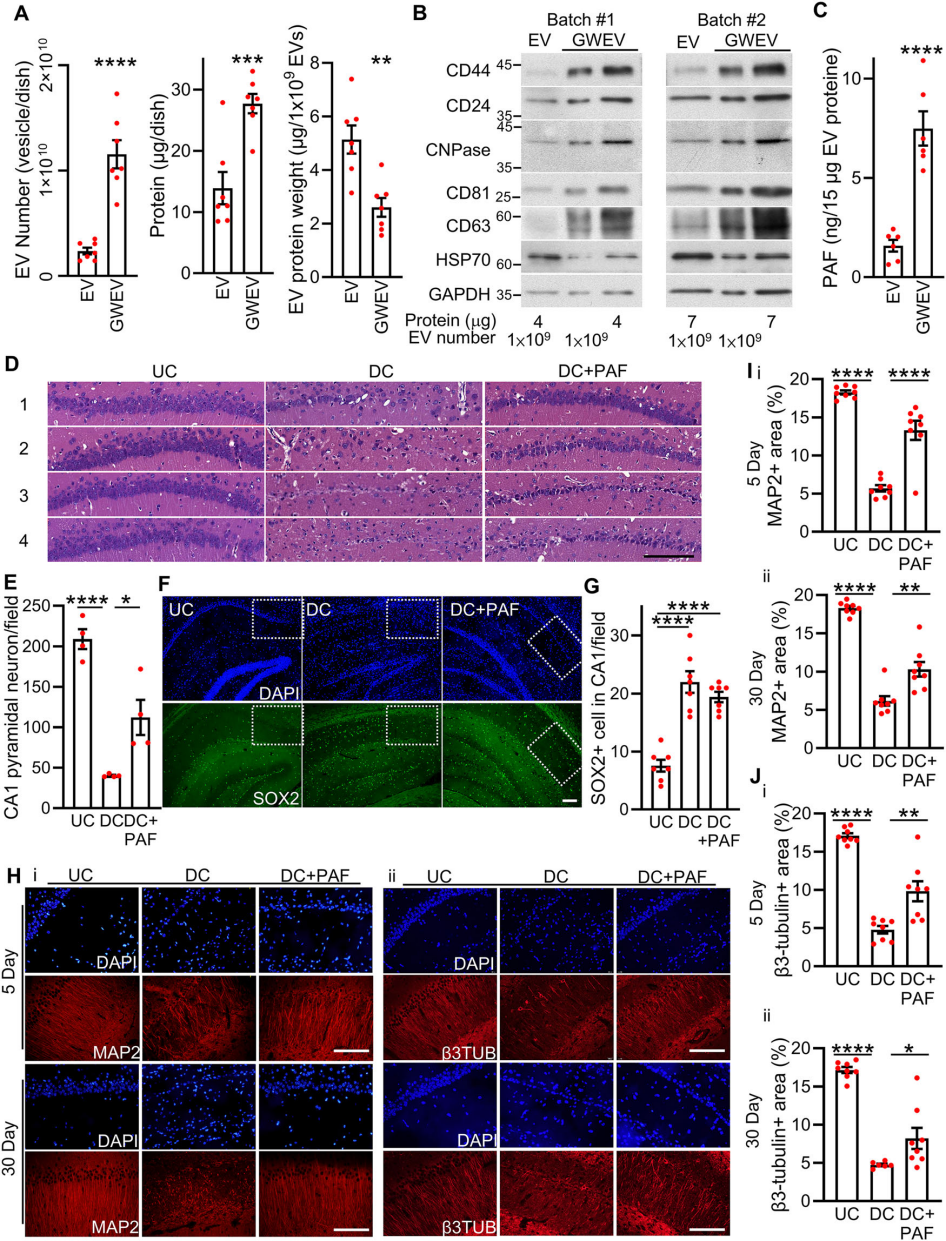
**EP4 inhibition induces EV enriched in PAF, promoting neuronal restoration in the CA1 region of injured hippocampi**. (A) Quantification of total EV number (left), total protein content (middle) and protein weight per 1 × 10^9^ EVs (right) from untreated MSCs (EV) and GW627368X‐treated MSCs (GWEV). Data are presented as mean ± SEM (*n* = 7). ***p ≤ 0.001; ****p ≤ 0.0001. (B) Western blot analysis comparing protein composition between EV and GWEV preparations, normalized either to total protein or to EV number. (C) ELISA quantification of PAF levels in EV and GWEV samples. Data are mean ± SEM (*n* = 6). ****p ≤ 0.0001. (D) Haematoxylin and eosin staining of the CA1 region in undamaged control (UC), PBS‐treated damaged control (DC) and PAF‐treated DC mice (DC+PAF). Scale bar: 100 µm. Field area: 51,776 µm^2^. (E) Quantification of CA1 pyramidal neurons in the hippocampi shown in panel D. Data represent mean ± SEM (*n* = 4 mice per group). *p ≤ 0.05; ****p ≤ 0.0001. (F) Immunofluorescence staining of SOX2, a marker of neural stem cells (NSCs), in the CA1 regions (white box) of hippocampi from UC, DC and DC+PAF mice 5 days post‐treatment. Nuclei were counterstained with DAPI. Scale bar: 100 µm. (G) Quantification of SOX2‐positive cells in CA1 regions corresponding to panel F. Data are mean ± SEM (*n* = 7 mice per group). ****p ≤ 0.0001. Field area: 62,500 µm^2^. (H) Immunofluorescence staining of microtubule‐associated protein 2 (MAP2, panel i) and neuronal microtubule element β3‐tubulin (β3TUB, panel ii) in the CA1 region of hippocampi from UC and DC mice at 5 and 30 days post‐treatment. Nuclei were stained with DAPI. Scale bar: 100 µm. (I) Quantification of MAP2 expression in the CA1 region corresponding to the representative images in panel H‐i. Panel I‐i shows data from 5 days post‐treatment, and panel I‐ii shows data from 30 days post‐treatment. Data are mean ± SEM (*n* = 8 mice per group). **p ≤ 0.01, ****p ≤ 0.0001. (J) Quantification of β3‐tubulin expression in the CA1 region corresponding to the representative images in panel H‐ii. Panel J‐i shows data from 5 days post‐treatment, and panel J‐ii shows data from 30 days post‐treatment. Data are mean ± SEM (*n* = 8 mice per group). *p ≤ 0.05, **p ≤ 0.01, ****p ≤ 0.0001.

Leveraging our previous report that defined stimulatory cues could prime MSCs to release EVs with specific cargo, we addressed both the yield and uniformity of the EV population (Figure S1A) (Chen et al. 2019, 2020). Herein, we demonstrated that the inhibition of prostaglandin E2 (PGE_2_) signalling through the EP4 receptor antagonist GW627368X resulted in a greater abundance of secreted EVs (Figure 1A, left panel) and an elevated total protein yield (Figure 1A, middle panel). These EVs are hereafter referred to as GWEVs.

Because the protein weight of EVs and GWEVs differed significantly (Figure 1A, right panel), we compared their cargo normalized either by total protein or by vesicle count. Western blot analyses revealed that several functionally relevant proteins, including CD44, CD24 and CNPase, in addition to classical EV markers CD81 and CD63, were preferentially enriched in GWEVs (Figure 1B). This enrichment was consistent whether normalized to total protein or vesicle number, relative to EVs from untreated MSCs. These proteins are known contributors to tissue repair and immune modulation: CD24 modulates systemic inflammation and supports immune homeostasis post‐injury (Wang et al. 2022; Liu and Zheng 2023), while CD44 facilitates infarct repair by regulating inflammatory and fibrotic responses (Huebener et al. 2008). Notably, CNPase in GWEVs can promote β3‐tubulin polymerization in neurons, playing an important role in cytoskeletal stabilization and neural regeneration (Chen et al. 2019, 2020). These findings suggest that EP4 antagonism not only enhances EV production but also selectively enriches vesicle cargo with proteins linked to tissue regeneration.

We then hypothesized that the sorting of metabolic cargo could also be affected. We performed mass spectrometry–based metabolomic profiling of GWEVs versus naive EVs. Differences in metabolite composition were observed across both organic and aqueous phases, analysed under positive and negative electrospray ionization (ESI) modes (Figure S1B). Seven potential metabolites were found to be elevated in GWEVs, with PAF exhibiting the most significant increase, 11‐ to 13‐fold higher abundance than naïve EVs (Figure S1C). Subsequent ELISA validation across multiple independent EV preparations confirmed that GWEVs contained approximately five times more PAF than EVs from untreated MSCs (Figure 1C). Together, these findings demonstrate that EP4 receptor inhibition enhances both EV biogenesis and the functional enrichment of bioactive lipids such as PAF, positioning GWEVs as a potent platform for regenerative delivery.

### 3.2 PAF Promotes Restoration of Hippocampal CA1 Pyramidal Neurons

We previously reported that systemic administration of GWEVs improves cognition, learning and memory in mice with hippocampal injury (Chen et al. 2019, 2020). To examine whether these therapeutic effects were derived from specific bioactive components within GWEVs, we then focused on the evaluation of the regenerative potential of PAF in a transgenic mouse model featuring inducible, CA1‐specific neuronal ablation.

Camk2a‐tTA/tetO‐DTA double‐transgenic mice express diphtheria toxin A (DTA) under the control of a doxycycline (Dox)‐regulated system. The tetracycline‐controlled transactivator (tTA), driven by the Camk2a promoter (Mayford et al. 1996, 1996; Sakaguchi et al. 2015), activates DTA expression in the forebrain, including the cerebral cortex and hippocampus, upon Dox withdrawal, leading to targeted neuronal damage. Previous studies have shown that Camk2a promoter‐driven transgene expression is strongest in CA1 neurons, but weaker in CA3 and the dentate gyrus (DG) (Mayford et al. 1996; Sakaguchi et al. 2015). Continuous Dox administration suppresses DTA expression, thereby preserving CA1 neurons, which are normally characterized by a densely packed pyramidal cell layer with extensive axonal arborization (Mizuseki et al. 2011). We analysed NeuN‐positive neurons across forebrain regions, including cortical regions and the hippocampus in Camk2a‐tTA/tetO‐DTA mice. Consistent with the strong Camk2a promoter activity in CA1, Dox withdrawal at 6 weeks of age induces DTA expression over 30 days (Figures S2A, B), resulting in substantial CA1 NeuN‐positive neuron loss (Figure S2C, arrowhead, and 2D‐i) compared with CA3 and DG (Figure S2C, 2D‐ii and 2D‐iii). In contrast, the Camk2a‐promoter‐mediated DTA expression did not cause significant loss of NeuN‐positive neurons in cortical regions of the forebrain (Figure S2C and 2D‐iv). After 30 days without Dox, Camk2a/DTA mice exhibited marked CA1 pyramidal neuron loss (Figures 1D,E and S2B; damaged control, DC), confirming effective lesioning. In contrast, single‐transgenic tetO‐DTA mice, which lack tTA expression, retained normal hippocampal architecture and served as undamaged controls (Figures 1D,E and S2B‐2D; undamaged control, UC).

To evaluate whether PAF potentiates neuronal recovery, DC mice were administered two intracardiac injections of PAF at the time points specified in Figure S2A. CA1 pyramidal neurons were quantified 5 days post‐treatment. PBS‐treated DC mice exhibited an 81% reduction in CA1 neuron numbers compared to UC mice (Figure 1D,E). In contrast, PAF‐treated DC mice demonstrated a significant restoration of CA1 neurons, reaching approximately 55% of baseline levels observed in UC mice (Figure 1D,E; DC+PAF). These results indicate that the lipid mediator PAF promotes partial restoration of hippocampal CA1 pyramidal neurons following injury, supporting its role as a key neuroregenerative effector.

### 3.3 PAF Enhances Neuritogenesis and Cytoskeletal Remodelling in the Damaged Hippocampus

To further explore the mechanism by which PAF promotes hippocampal neuron restoration, we investigated its effects on neural stem cells (NSCs) in the context of injury. SOX2 serves as a key transcription factor maintaining NSC identity and self‐renewal (Ferri et al. 2004; Avilion et al. 2003; Suh et al. 2007). We therefore examined whether PAF modulates the number of SOX2‐positive NSCs in hippocampal CA1 regions following injury. The number of SOX2‐positive cells in CA1 region was evaluated in undamaged DTA mice (UC), PBS‐treated Camk2a/DTA mice (DC), and PAF‐treated DC mice (DC+PAF). Compared to UC mice, both DC and DC+PAF groups exhibited elevated SOX2‐positive cells 5 days post‐injury (Figure 1F, G), consistent with an endogenous NSC response to damage. However, PAF treatment did not lead to an additional increase in SOX2‐positive NSCs, suggesting that PAF does not promote stem cell expansion in this context.

We next examined whether PAF facilitates the recovery of axonal projections of pyramidal neurons in the injured CA1 regions. To assess this, we analysed the expression of β3‐tubulin and microtubule‐associated protein 2 (MAP2) in the hippocampal sections from UC, DC, and DC+PAF mice, following the dosing regimen in Figure S2A. MAP2 is predominantly localized to neuronal processes and interacts with β3‐tubulin to promote and stabilize microtubule growth (Korzhevskii et al. 2012). Its expression is closely associated with the recovery of neuronal function following injury (Yamanouchi et al. 1998). Immunostaining revealed intact MAP2‐positive neurites in CA1 regions of both UC and DC+PAF mice 5 days post‐treatment, while DC mice showed sparse MAP2 labelling (Figure 1H‐i,I‐i). Quantitative analysis confirmed this: MAP2‐positive area in DC+PAF mice reached 13%, approaching the 18% observed in UC mice. Notably, MAP2 expression in DC+PAF mice remained stable at this level even 30 days post‐treatment (Figure 1H‐i,I‐ii), underpinning a sustained neuroregenerative effect exerted by PAF treatment.

To further examine PAF's impact on cytoskeletal remodelling, we analysed β3‐tubulin, a marker of microtubule assembly and early neurite extension. Five days post‐treatment, β3‐tubulin expression in CA1 was significantly increased in DC+PAF mice compared to PBS‐treated DC mice (Figure 1H‐ii,J‐i). In UC mice, β3‐tubulin‐positive neurites covered approximately 17% of the CA1 area; in contrast, DC mice showed a reduction to 5%, while PAF‐treated mice exhibited partial restoration to 10%. This increase in β3‐tubulin expression was also maintained 30 days post‐treatment (Figure 1H‐ii,J‐ii), suggesting that PAF promotes long‐term cytoskeletal stabilization. Collectively, these results show that PAF not only increases the number of CA1 pyramidal neurons, but also promotes microtubule stabilization and neurite outgrowth in CA1 regions.

This dual effect on structural regeneration highlights a central role for PAF in driving functional and morphological regeneration following hippocampal injury, strongly supporting that the therapeutic efficacy of GWEVs in neural repair is mediated with PAF treatment.

### 3.4 PAF Promotes Structural Maturation of Hippocampal Progenitors

**FIGURE 2:**
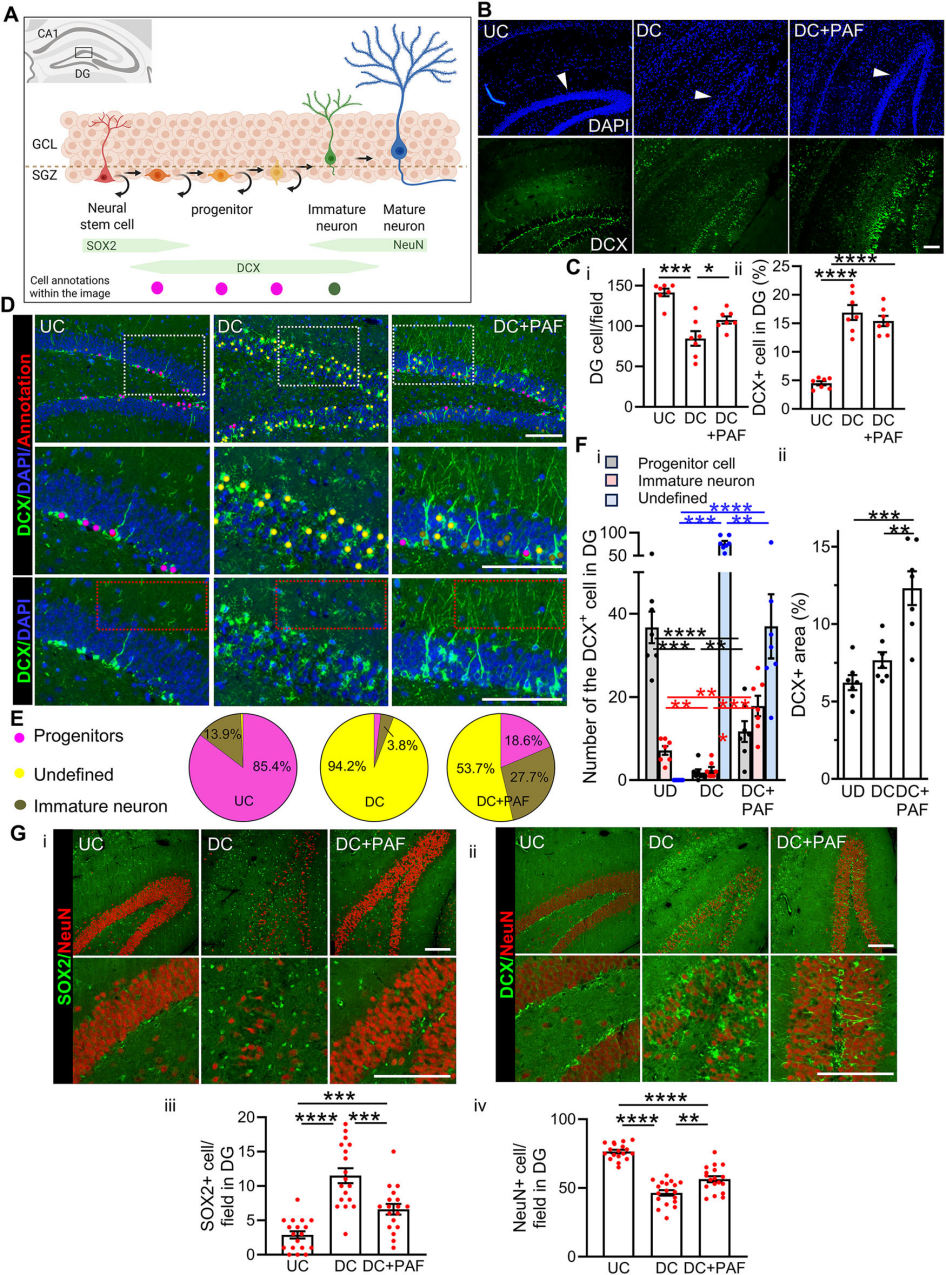
**PAF promotes progenitor maturation and neuritogenesis in dentate gyrus (DG) of damaged hippocampi**. (A) Illustration of adult neurogenesis in the DG. The inset (top left) shows the location of the DG within the hippocampus, adjacent to the cornu ammonis (CA) regions. The main schematic depicts sequential neuronal development stages, beginning with a radial glia‐like neural stem cell (red), progressing through progenitor proliferation and differentiation (orange to yellow), to immature neurons (green) and mature granule neurons (blue). Straight arrows indicate differentiation, while circular arrows represent self‐renewal. The green bars below mark the expression period of SOX2, doublecortin (DCX) and NeuN. The granule cell layer (GCL) contains both mature and immature neurons with extended neurites into the outer molecular layer neurites, whereas the subgranular zone (SGZ)—a key neurogenic niche—houses neural stem and progenitor cells. (B) Immunofluorescence staining of DCX, a marker of neural progenitors and immature neurons, in the DG (arrowhead) of UC, DC and DC+PAF mice 5 days post‐treatment. Nuclei were counterstained with DAPI. Scale bar: 100 µm. (C) Quantification of total cells (panel i) and DCX‐positive cells (panel ii) in the DG of mice shown in panel B. Data are mean ± SEM (*n* = 7 mice per group). ****p ≤ 0.0001. Field area: 6,645 µm^2^. (D) Immunofluorescence staining of DCX in the DG of UC, PBS‐treated damaged (DC), and PAF‐treated damaged (DC+PAF) mice, 5 days post‐treatment. Nuclei were stained with DAPI. DCX‐positive cell types were annotated based on cell morphology and spatial position. Middle and bottom panels show higher‐magnification views of the white dashed areas in the top panels. Scale bar: 100 µm. (E) Quantification of DCX‐positive cell types classified by morphology and spatial location, as illustrated in panel A. Pie charts shows proportions of DCX‐positive cells categorized as progenitors (magenta), immature neurons (olive green), or undefined (yellow) in the DG of UC, DC and DC+PAF mice (*n* = 7 mice per group). (F) Quantification of DCX‐positive cells in DGs corresponding to the representative images in panel D. Panel i shows the number of each DCX‐positive cell type; panel ii shows the DCX‐positive area in the outer molecular layer (red dashed rectangles). Data are mean ± SEM (*n* = 7 mice per group). **p ≤ 0.01; ***p ≤ 0.001; ****p ≤ 0.0001. (G) Immunofluorescence staining of SOX2 (panel i), NeuN (Panel i and ii), and DCX (Panel ii) in the DGs of hippocampi from UC, DC, and DC+PAF mice at 5 days post‐treatment. Scale bar: 100 µm. Quantification of SOX2‐positive (panel iii) and NeuN‐positive (panel iv) cells in the DGs is shown. Data are mean ± SEM (*n* = 18; three fields per mouse for six mice). **p ≤ 0.01, ***p ≤ 0.001, ****p ≤ 0.0001. Field area: 8,000 µm^2^.

In the adult brain, neurogenesis is primarily restricted to two regions: the DG of the hippocampus and the subventricular zone adjacent to the lateral ventricles (Gage and Temple 2013). These niches contain NSCs and neuron progenitor cells (NPCs) capable of generating new neurons throughout life. In the DG, NSCs in the subgranular zone (SGZ) exhibit triangular somas and radial processes spanning the granule cell layer (GCL). Upon differentiation, these cells lose their radial morphology and begin expressing Doublecortin (DCX), a microtubule‐associated protein and established marker of NPCs and immature neurons (Figure 2A) (Couillard‐Despres et al. 2005). DCX expression reflects neurogenic activity and is sensitive to age and neurological insults (D'Alessio et al. 2010, Plümpe et al. 2006, Rao and Shetty 2004).

In DC mice, PAF treatment partially restored DG cellularity, as reflected by increased cell density (Figure 2B,C‐i; DC vs. DC+PAF). To determine whether PAF influences neuronal differentiation in the DG after injury, we examined DCX expression in this region. In UC mice, DCX‐positive cells were localized primarily within the SGZ, the site of active neurogenesis (Figure 2B, UC). In contrast, both PBS‐ and PAF‐treated DC mice exhibited similarly elevated proportions of DCX‐positive cells 5 days post‐treatment (Figure 2B,C‐ii), indicating that hippocampal injury itself upregulated DCX expression in the DG, and systemic PAF administration did not further increase the overall number of DCX‐positive cells.

In UC mice, DCX‐positive cells were largely restricted to the SGZ, consistent with their progenitor identity (Figure 2A,D, UC) (Rolando and Taylor 2014; Nicola et al. 2015). In contrast, in both DC and DC+PAF mice, DCX‐positive cells extended into the GCL (Figure 2A,D, DC and DC+PAF). Within the GCL, immature neurons typically exhibit bipolar or pyramidal morphology with short neurites, while mature neurons display larger somas and complex arborization extending into the molecular layer (Figure 2A). Based on morphological and spatial characteristics, DCX‐positive cells were classified as progenitors (magenta), immature neurons (olive green), or undefined cells (yellow) that did not fit either category (Figures 2A,D and S3). In UC mice, 85% of DCX‐positive cells were classified as progenitors and 14% as immature neurons (Figure 2D,E, left). In DC mice, 94% of DCX‐positive cells exhibited atypical or undefined cellular morphology (Figure 2D,E, middle). Strikingly, in DC+PAF mice, the undefined population decreased to 54%, accompanied by an increase in progenitors (from 2% to 19%) and immature neurons (from 4% to 28%) (Figure 2D,E, right). Overall, PAF treatment led to a 7.5‐fold increase in DCX‐positive immature neurons within the DG (Figure 2F‐i, pink bar; DC vs. DC+PAF).

Beyond serving as a neurogenesis marker, DCX stabilizes microtubules and promotes neurite extension during early neuronal development (Gleeson et al. 1999, Friocourt et al. 2003, Moon and Wynshaw‐Boris 2013). To assess whether PAF enhances neurite maturation of DCX‐positive cells, we analysed DCX‐labelled neurites extending into the outer molecular layer (Figure 2D, red box). Robust DCX‐positive neurites were observed in DC+PAF mice 5 days post‐treatment, while UC and DC mice showed sparse DCX labelling (Figure 2D, red box and Figure S3). Quantification confirmed this increase: DCX‐positive area in outer molecular layers reached 12% in DC+PAF mice compared with 6% in UC mice (Figure 2F‐ii).

The concurrent expansion in DCX‐positive cells within the GCL and their extended neurites into the outer molecular layer in DC+PAF mice indicates that PAF promotes structural maturation of DG progenitors following injury. To further assess whether this differentiation alters other neuronal populations, we quantified SOX2‐positive NSCs and NeuN‐positive mature neurons in the DG of UC, DC and DC+PAF mice (Figure 2G). Compared with UC mice, DC mice exhibited a significant increase in SOX2‐positive cells in the DGs 5 days post‐injury (Figure 2G‐i,G‐iii), consistent with an endogenous NSC proliferative response after injury. PAF treatment reduced the number of SOX2‐positive cells in DC mice (Figure 2G‐i,G‐iii), suggesting that PAF promotes NSC differentiation, thereby depleting the pool of undifferentiated SOX‐2 positive cells in the DG. Conversely, DC mice showed a significant reduction in NeuN‐positive mature neurons relative to UC mice (Figure 2G‐ii,G‐iv), reflecting neuronal loss, while PAF treatment restored NeuN‐positive cell numbers, indicating enhanced neuronal replenishment through differentiation and maturation of DCX‐positive progenitors.

In summary, these results identify PAF as a key regulator of post‐injury neurogenesis in the DG. PAF drives the structural maturation of DCX‐positive progenitors into immature and mature neurons, accompanied by enhanced neuritogenesis and a reduction in undifferentiated SOX2‐positive NSCs. The replenishment of NeuN‐positive neurons further supports a role for PAF in functional neuronal replacement following hippocampal damage. Together, these findings highlight PAF as a potent modulator of progenitor maturation and neurite remodelling in the adult hippocampus.

### 3.5 Neuronal Regeneration and Neuritogenesis by PAF Require Metabolic Processing by PAFAH

**FIGURE 3:**
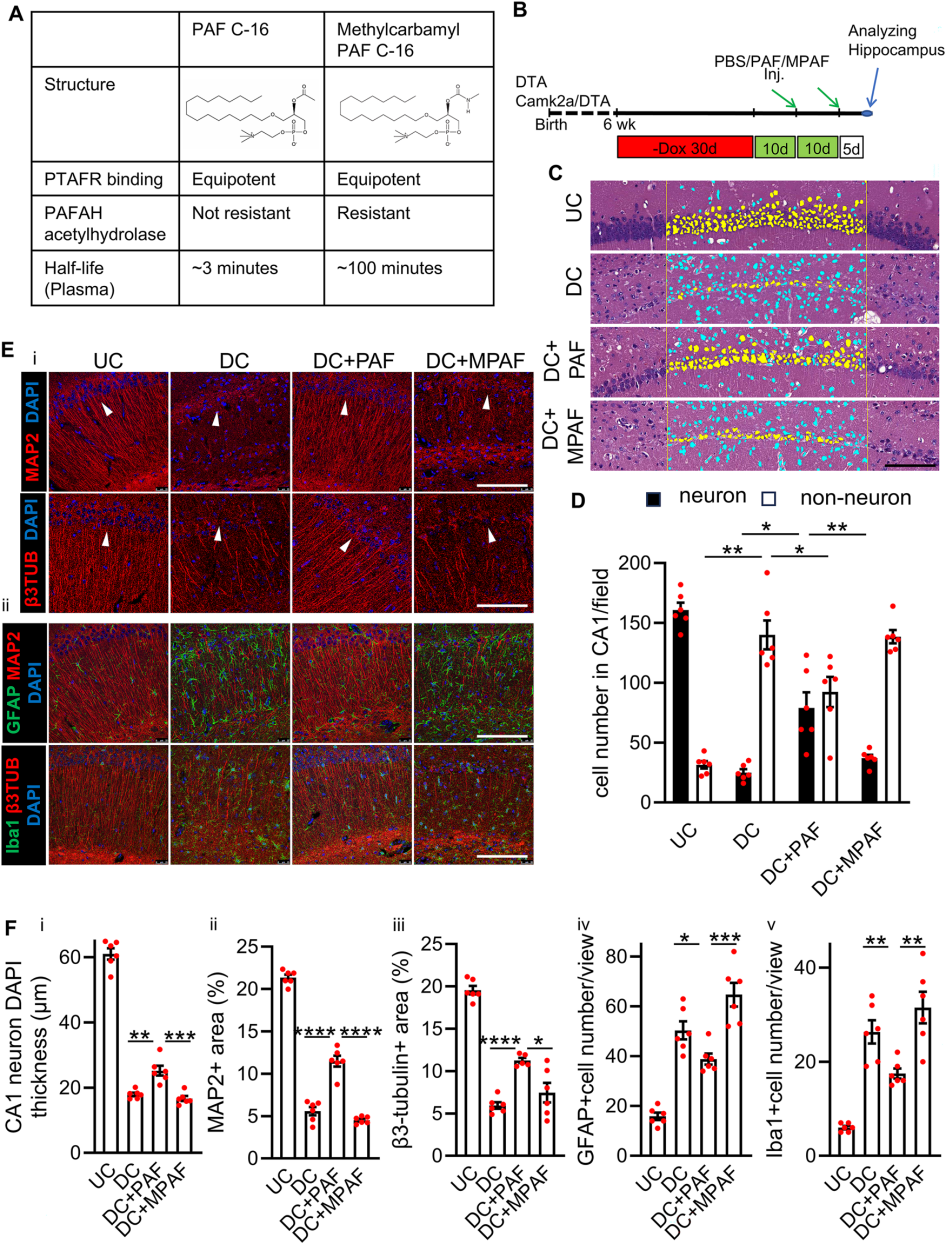
**PAF promotes neuroregeneration while suppressing astrogliosis and neuroinflammation in injured brains, effects not reproduced by MPAF**. (A) Comparison of PAF and methylcarbamyl PAF (MPAF), a stable analogue of PAF. (B) Schematic of the experimental timeline showing induction of hippocampal damage, administration of PAF or MPAF and tissue collection. (C) Haematoxylin and eosin staining of the CA1 region in hippocampi from undamaged control (UC) and damaged control (DC) mice, 5 days post‐treatment with PBS (DC), PAF (DC+PAF), or MPAF (1.5 µg/kg, IC; DC+MPAF). Cells within the marked area were classified using QuPath based on morphology: pyramidal neurons (yellow), non‐neuronal cells (blue). Scale bar: 100 µm. (D) Quantification of CA1 pyramidal neurons and non‐neuronal cells from panel C. Data represent mean ± SEM (*n* = 6 mice per group). *p ≤ 0.05, **p ≤ 0.01, ***p ≤ 0.001. Field area: 59,947 µm^2^. (E) Representative immunofluorescence images of the CA1 region from UC, DC, DC + PAF and DC + MPAF mice, 5 days post‐treatment. Images show MAP2 (red; upper panels of i and ii), β3‐tubulin (β3TUB; red; bottom panels of i and ii), the astrocyte marker GFAP (green; upper panels of ii), and the microglial marker Iba1 (green; bottom panels of ii). Nuclei were counterstained with DAPI (blue). Arrowheads indicate the location of the CA1 pyramidal neuron soma layer. Scale bar, 100 µm. (F) Quantification of CA1 neuronal layer thickness, MAP2 and β3TUB expression, and numbers of GFAP‐ and Iba1‐positive cells in mice corresponding to the representative images in panel E. Panel i, CA1 neuronal layer thickness (quantification of E‐i, blue); Panel ii, MAP2 expression (quantification of E‐i and ii, upper panels, red); Panel iii, β3TUB expression (quantification of E‐i and ii, bottom panels, red); Panel iv, GFAP‐positive cell counts (quantification of E‐ii, upper panels, green); and Panel v, Iba1‐positive cell counts (quantification of E‐ii, bottom panels, green);. Data represent mean ± SEM (*n* = 6 mice per group). *p ≤ 0.05, **p ≤ 0.01, ***p ≤ 0.001, ****p ≤ 0.0001. Field area: 60,516 µm^2^.

We then hypothesized that PAF's neuroregenerative effects are mediated through its receptor signalling. PAF is a potent lipid signalling molecule involved in cell growth, differentiation and inflammation, primarily acting via G protein‐coupled PAF receptors (PTAFR) (Kume and Shimizu 1997). On the other hand, PAF signalling is tightly regulated by PAF‐acetylhydrolase (PAFAH), which inactivates PAF through deacetylation (Arai et al. 2002). To dissect the contribution of receptor signalling versus metabolic regulation, we compared native PAF with a degradation‐resistant analogue, methylcarbamyl PAF (MPAF). MPAF retains full PTAFR agonist activity but cannot be hydrolysed by PAFAH, resulting in a plasma half‐life exceeding 100 min (Figure 3A) (Kume and Shimizu 1997).

We administered PAF, MPAF, or PBS via intracardiac injection to hippocampally injured Camk2a/DTA mice and assessed CA1 pyramidal neuron recovery 5 days later (Figure 3B). CA1 pyramidal neurons were identified using QuPath classifier, based on nuclear morphology, including triangular somas, large nuclei and prominent nucleoli (Figure 3C, yellow). Compared to undamaged control (UC) mice, PBS‐treated damaged control (DC) mice exhibited an 84% reduction in CA1 pyramidal neurons, along with a reciprocal increase in non‐neuronal cells (Figure 3C, blue; Figure 3D). PAF treatment significantly restored neuronal numbers, achieving ∼50% of UC levels (Figure 3D, DC+PAF). In contrast, MPAF elicited only a modest and non‐significant increase (Figure 3D, DC+MPAF). These results were corroborated by the analysis of soma layer thickness, which was preserved in PAF‐ but not MPAF‐treated mice (Figure 3E‐i, arrowhead, nuclei labelled with DAPI in blue; and 3F‐i). To assess neuritogenesis, we evaluated MAP2 expression, a marker of dendritic structure. In UC mice, MAP2‐positive neurites accounted for ∼21% of the CA1 area, which dropped to 6% in DC mice (Figure 3E‐i, upper panels, MAP2 shown in red; 3F‐ii). PAF treatment restored MAP2 expression to 11%, while MPAF had no significant effect, mirroring PBS‐treated controls. We next assessed β3‐tubulin, a key component of microtubules involved in early neurite extension. In UC mice, β3‐tubulin‐positive neurites covered 20% of the CA1 area, which declined to 6% in DC mice. PAF elevated this to 11%, whereas MPAF again showed no measurable benefit (Figure 3E‐i, bottom panels, β3‐tubulin shown in red; 3F‐iii).

Taken together, these data demonstrate that PAF promotes neuronal survival and neurite regeneration in the damaged hippocampus. The inability of MPAF to replicate PAF's therapeutic effect suggests that in addition to the pathway activation, metabolic processing by PAFAH plays an important role in regenerative bioactivity. These findings support a PAFAH‐dependent, microtubule‐specific mechanism underlying the therapeutic efficacy of PAF in hippocampal repair.

### 3.6 PAF Suppresses Astrogliosis and Microglial Activation via PAFAH‐Dependent Processing

In addition to pyramidal neuron loss in the CA1 region, hippocampal injury was accompanied by a marked increase in non‐neuronal cell populations (Figure 3C, blue; Figure 3D). This elevation was significantly suppressed by PAF treatment but remained unchanged in MPAF‐treated mice (Figure 3D). This elevation, typically associated with gliosis, was significantly reduced by PAF treatment, but remained unchanged in mice treated with the degradation‐resistant analogue MPAF (Figure 3D). Reactive astrogliosis and microglial activation are hallmark responses to CNS injury and are strongly linked to neuroinflammation and impaired recovery (Pekny and Pekna 2014, 2016; Lucas et al. 2006). To determine whether PAF modulates these injury‐induced glial responses, we examined the expression of glial fibrillary acidic protein (GFAP) and ionized calcium‐binding adapter molecule 1 (Iba1), canonical markers for astrocytes and microglia, respectively, in the CA1 region. Following 30 days of Dox withdrawal to induce selective CA1 neuron ablation, DC mice received two intracardiac injections of PBS, PAF, or MPAF. Five days post‐treatment, hippocampal tissue was collected for immunohistochemical analysis. Compared to undamaged control (UC) mice, DC mice exhibited a threefold increase in GFAP‐positive astrocytes, indicating severe astrogliosis (Figure 3E‐ii, upper panels; 3F‐iv). PAF treatment reduced the number of GFAP‐positive astrocytes by 24% relative to PBS‐treated controls. In contrast, MPAF not only failed to attenuate GFAP expression, but appeared to exacerbate astrocyte activation. A similar pattern was observed for microglial infiltration. The number of Iba1‐positive microglia was fourfold higher in DC mice relative to UC controls (Figure 3E‐ii, bottom panels; 3F‐v). PAF administration significantly decreased Iba1‐positive microglial numbers by 35%, indicating suppression of microglial activation. Again, MPAF treatment had no beneficial effect and appeared to worsen microglial reactivity.

These findings demonstrate that PAF attenuates both astrogliosis and microglial activation in the injured hippocampus, likely through mechanisms requiring enzymatic processing by PAFAH. The inability of MPAF to reduce glial activation underscores the essential role of PAF metabolism in mediating these anti‐inflammatory effects. Together, these results expand the therapeutic profile of PAF, showing that its neuroprotective actions extend beyond neuronal regeneration to include modulation of glial reactivity and immune cell infiltration, two key contributors to secondary damage and impaired recovery following CNS injury.

### 3.7 Cognitive Recovery by PAF Requires PAFAH‐Mediated Processing

**FIGURE 4:**
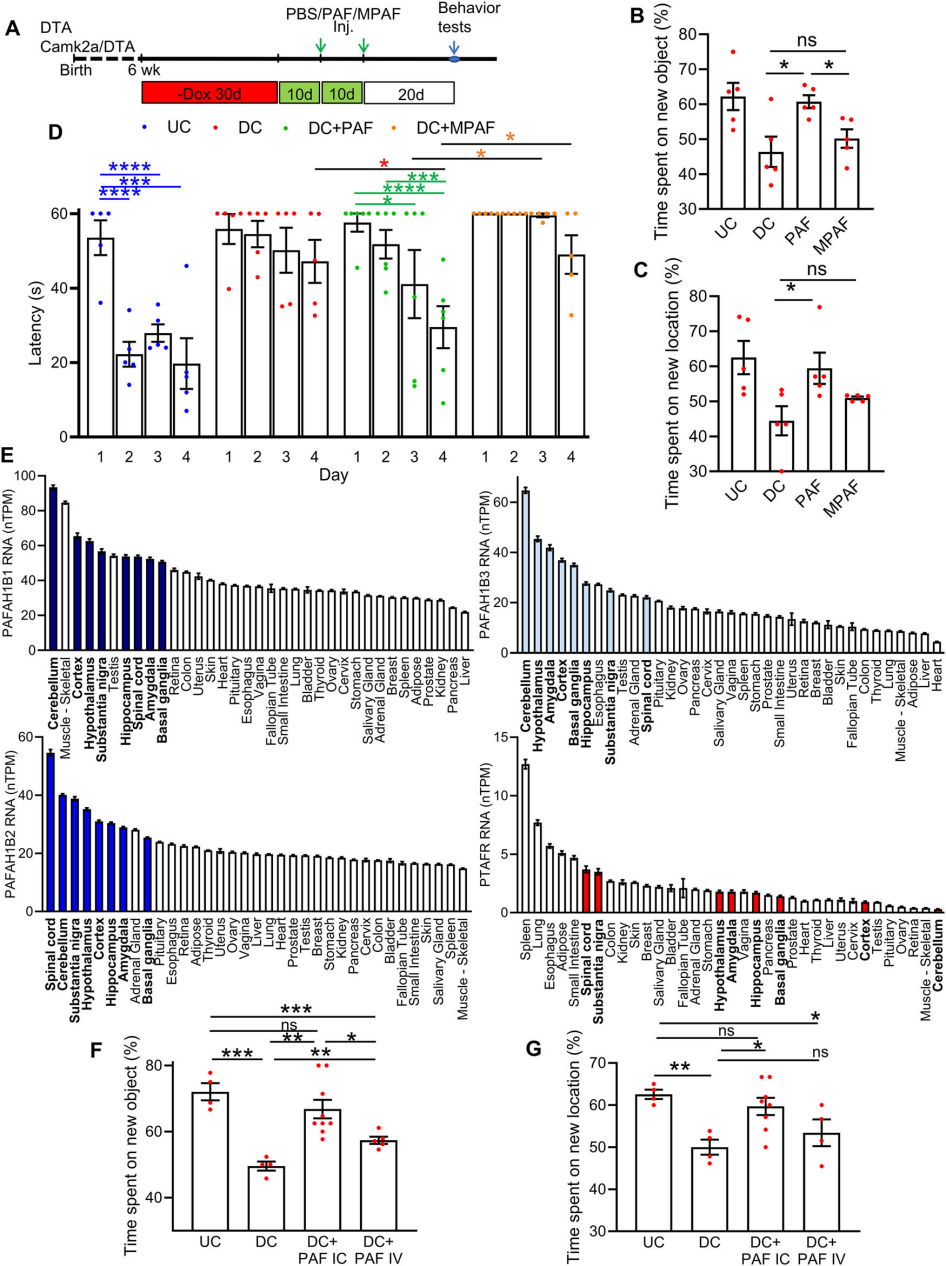
**PAF restores cognitive function after hippocampal injury, an effect not reproduced by MPAF**. (A) Schematic of the experimental timeline for hippocampal damage induction, treatment with PAF or methylcarbamyl PAF (MPAF), and subsequent behavioural testing. (B, C) Novel Object Recognition Test (NORT) and Novel Location Recognition Test (NLRT): Percentage of time spent exploring the novel object (B) or location (C) in UC and DC mice, 30 days after treatment with PBS (DC), PAF (DC+PAF), or MPAF (DC+MPAF). Data are mean ± SEM (*n* = 5 mice per group). *p ≤ 0.05. (D) Morris Water Maze (MWM): Latency to locate the hidden platform across training days in UC and DC mice treated as in (B). Data are mean ± SEM (*n* = 5 mice per group). *p ≤ 0.05, ***p ≤ 0.001, ****p ≤ 0.0001. (E) Tissue‐specific expression of PAFAH1B1, PAFAH1B2, PAFAH1B3 and PTAFR mRNA across human organs, derived from GTEx RNA‐seq data (dbGaP Accession phs000424.v8.p2). Data are expressed in transcripts per million (TPM) and shown as mean ± SEM. ****p ≤ 0.0001. (F, G) Effect of administration route on memory rescue: Percentage of time spent exploring the novel object (F) and location (G) in UC and DC mice treated with PBS (DC), intracardially delivered PAF (DC+PAF IC), or intravenously delivered PAF (DC+PAF IV). Data are mean ± SEM (*n* = number of spots as mice per group). *p ≤ 0.05.

Given the critical role of the hippocampus in memory consolidation and spatial navigation (Clark et al. 2005), we investigated whether PAF could restore cognitive function following hippocampal injury. After Dox withdrawal, mice received two systemic injections of PBS, PAF, or MPAF at the time points indicated in Figure 4A. Behavioural testing was conducted 20 days post‐treatment using the novel location recognition test (NLRT), novel object recognition test (NORT), and the Morris water maze (MWM) (Figure 4A).

The NLRT and NORT assess recognition memory based on rodents’ innate preference for novelty (Mumby et al. 2002). Undamaged control (UC) mice spent ∼62% of their exploration time on the novel object or in the novel location, indicating intact memory (Figure 4B,C). In contrast, PBS‐injected DC mice spent nearly equal time on novel and familiar objects or locations, showing no preference for novelty. This reflects impaired recognition memory due to CA1 neuron loss. MPAF‐injected DC mice exhibited similar deficits, confirming its lack of efficacy (Figure 4B,C). Strikingly, PAF‐treated DC mice displayed a significant preference for novel stimuli, spending ∼60% of their exploration time on the novel object/location, comparable to UC mice (Figure 4B,C). These results demonstrate that PAF, but not MPAF, restored recognition memory after hippocampal damage.

To assess spatial learning, we used the MWM (Morris 1984). Mice were trained to locate a submerged platform using spatial cues. UC mice showed progressive reductions in escape latency, consistent with effective spatial memory acquisition (Figure 4D, blue). PBS‐ and MPAF‐treated DC mice failed to improve across testing days, indicating persistent learning impairments (Figure 4D, red and orange). In contrast, PAF‐treated DC mice demonstrated significant improvement, with escape latencies decreasing by Day 3 and Day 4 (Figure 4D, green), indicating recovery of spatial learning.

Together, these behavioural findings show that PAF effectively rescues both recognition and spatial memory deficits induced by hippocampal CA1 damage. The inability of MPAF to confer similar benefits underscores the importance of PAF's metabolic processing via PAFAH in mediating its cognitive effects.

### 3.8 Therapeutic Efficacy of PAF Depends on Brain‐Localized PAFAH and CNS Access

PAF has been shown to reverse pathological alterations and restore recognition and spatial memory deficits caused by hippocampal injury. The failure of MPAF, a degradation‐resistant analogue, to confer similar benefits underscores the critical role of PAFAH in mediating PAF's therapeutic effects. PAFAH is a heterotrimeric enzyme complex composed of two catalytic subunits (PAFAH1B2, PAFAH1B3) and a regulatory subunit (PAFAH1B1). Notably, PAFAH1B1, encoded by the LIS1 gene, is essential for neuronal migration and microtubule organization, and its mutation is associated with Type I lissencephaly (Arai et al. 2002; Shmueli et al. 1999). To investigate the relationship between PAFAH expression and brain‐specific therapeutic outcomes, we analysed RNA‐seq data from the GTEx database, focusing on PAFAH1B1, PAFAH1B2, PAFAH1B3 and PTAFR across multiple human tissues. Among all tissues, the highest expression of PAFAH subunits was observed in the brain (Figure 4E, blue), indicating strong neuronal enrichment. In contrast, PTAFR expression was low in the brain and highest in the spleen (Figure 4E, red), suggesting that PAF's effects in the CNS are likely mediated through enzymatic metabolism by PAFAH, rather than via classical receptor signalling.

To determine whether PAF's therapeutic efficacy depends on direct brain access, we compared intravenous (IV) and intracardiac (IC) delivery in hippocampal‐injured mice. Although IV injection is subject to pulmonary first‐pass sequestration (Leibacher and Henschler 2016, Lee et al. 2009), IC delivery allows broader systemic and cerebral distribution (Scarfe et al. 2018). Behavioural testing via the NORT and NLRT revealed that IC‐administered PAF restored recognition memory: DC+PAF (IC) mice spent ∼67% and ∼60% of their exploration time on the novel object and location, respectively, not significantly different from UC mice (Figure 4F,G, DC+PAF IC vs. UC). In contrast, DC+PAF (IV) mice spent ∼57% and ∼53% of their exploration time on the novel object and location, significantly lower than UC mice (Figure 4F,G, DC+PAF IV vs. UC). Thus, IC‐, but not IV‐, PAF delivery restored memory performance to undamaged control levels. These findings indicate that effective brain delivery of PAF is essential for therapeutic benefit, and that its neuroregenerative effects depend on metabolic processing by PAFAH within neural tissue. Targeted delivery strategies, such as intracardiac administration, may be critical for leveraging PAF's full therapeutic potential in CNS repair.

### 3.9 Neuronal PAFAH Mediates the Neuritogenic Effects of PAF

**FIGURE 5:**
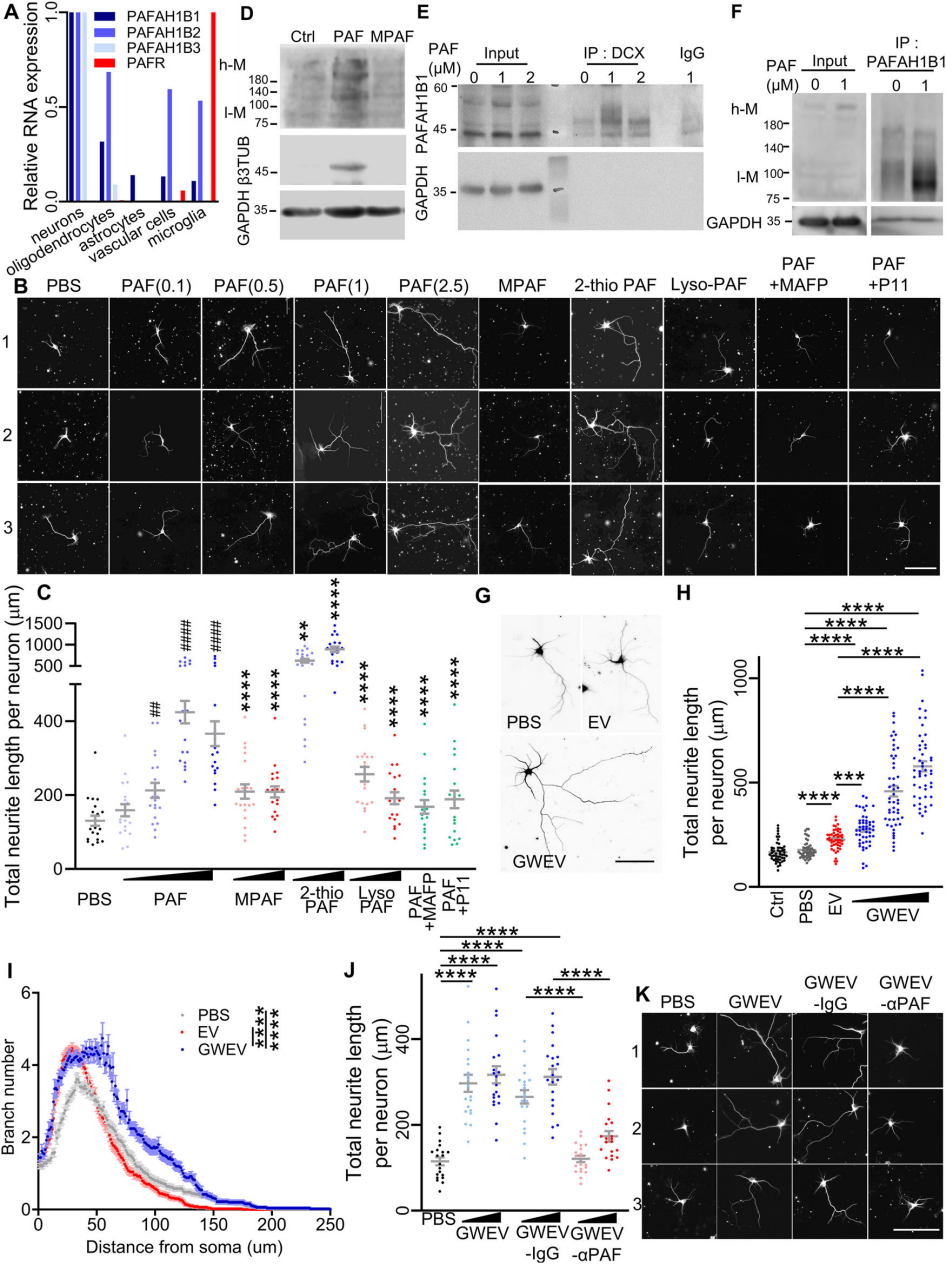
**PAF‐enriched GWEVs stimulate neuritogenesis in hippocampal pyramidal neurons through PAFAH–mediated PAF metabolism**. (A) Expression profiles of PAFAH1B1, PAFAH1B2, PAFAH1B3 and PTAFR across major brain cell types, derived from spatial transcriptomic data. Data represent relative RNA expression values (Neuroscience Multi‐omics Archive, RRID:SCR_01615). (B) Representative β3‐tubulin immunostaining of primary hippocampal neurons cultured for 3 days with PBS, PAF (0.1, 0.5, 1, and 2.5 µM), MPAF (1 and 5 µM), 2‐thio‐PAF (1 and 5 µM), lyso‐PAF (1 and 5 µM), PAF (1 µM) + MAFP (500 nM), or PAF (1 µM) + P11 (37 nM). Scale bar: 100 µm. (C) Quantification of total neurite length in hippocampal neurons described in (B). Data are mean ± SEM (*n* = 20 neurons per group). ^##^ *p* ≤ 0.01, ^####^ *p* ≤ 0.0001, compared with PBS‐treated group; ****p ≤ 0.0001, compared with PAF (1 µM). (D) Western blot analysis of cytoskeletal proteins β3‐tubulin (β3TUB) and MAP2 in NE‐4C cells treated with PBS (control), PAF (1 µM), or MPAF (1 µM) for 48 h. Both high‐molecular‐weight MAP2 isoforms (MAP2A/B, ∼280 kDa; h‐M) and low‐molecular‐weight isoforms (MAP2C/D, ∼75 kDa; l‐M) are detected. (E) Co‐immunoprecipitation demonstrating enhanced interaction between endogenous PAFAH1B1 and DCX in NE‐4C cells after PAF treatment. DCX was immunoprecipitated, and co‐associated PAFAH1B1 was detected. Normal IgG served as a negative control. (F) Co‐immunoprecipitation showing interaction between PAFAH1B1 and MAP2 in NE‐4C cells treated with PBS or PAF. PAFAH1B1 was immunoprecipitated with anti‐PAFAH1B1 antibodies, and co‐precipitated MAP2 isoforms were detected (h‐M: MAP2A/B; l‐M: MAP2C/D). Normal IgG was used as a negative control. (G) Representative β3‐tubulin immunostaining of primary hippocampal neurons cultured with PBS, naïve EVs (EV, 3 × 10^8^ vesicles/well), or GWEVs (1 × 10^8^, 3 × 10^8^, 4 × 10^8^ vesicles/well) for 3 days. Scale bar: 100 µm. (H) Quantification of total neurite length in hippocampal neurons described in (G). Data are mean ± SEM (*n* = 50 neurons per group). ***p ≤ 0.001, ****p ≤ 0.0001. (I) Sholl analysis of neurite arborization in neurons described in (G). Data are shown as mean ± SEM (*n* = 50 neurons per group). ****p ≤ 0.0001. (J) Representative β3‐tubulin immunostaining of primary hippocampal neurons cultured with PBS, GWEVs (1 × 10^8^ or 2 × 10^8^ vesicles/well), IgG‐loaded GWEVs (GWEV‐IgG), or anti‐PAF antibody‐loaded GWEVs (GWEV‐αPAF) for 3 days. Scale bar: 100 µm. (K) Quantification of total neurite length in hippocampal neurons described in (J). Data are mean ± SEM (*n* = 20 neurons per group). ****p ≤ 0.0001.

To identify the principal cellular targets of PAF in the brain, we analysed spatial transcriptomics data from the Neuroscience Multi‐omics Archive (RRID:SCR_01615), focusing on all three PAFAH subunits (PAFAH1B1, PAFAH1B2, PAFAH1B3) and PTAFR across major brain cell types. Neurons exhibited the highest expression of all three PAFAH subunits (Figure 5A, blue), indicating strong enrichment of PAF metabolic activity in neuronal populations. In contrast, PTAFR expression was predominantly localized to microglia and was low in neurons (Figure 5A, red), suggesting that the therapeutic effects of PAF in the CNS are likely mediated through neuronal PAFAH, rather than classical receptor signalling.

To test the hypothesis, we examined the neuritogenesis‐promoting efficacy of a series of PAF analogues with distinct susceptibilities to hydrolysis by PAFAH (Deigner et al. 1999, Tsaoussis and Vakirtzi‐Lemonias 1994) and evaluated PAF function in the presence or absence of PAFAH inhibitors (Figure 5B,C) on primary hippocampal pyramidal neurons isolated at embryonic Day 17.5 (E17.5). The developmental progression of hippocampal neurons in culture is well characterized by distinct morphological stages (Figure S4). PAF induced neuritogenesis in primary hippocampal neurons in a dose‐dependent manner, with progressive increases in total neurite length observed at concentrations ranging from 1 to 2.5 µM (Figure 5B,C). We then compared the effects of two structural analogue, 2‐thio PAF, which is hydrolysed by PAFAH at a rate similar to native PAF (Tsaoussis and Vakirtzi‐Lemonias 1994; Liu et al. 2013), and MPAF, which is resistant to enzymatic hydrolysis (Deigner et al. 1999; Tsaoussis and Vakirtzi‐Lemonias 1994). Although 2‐thio PAF promoted neuritogenesis comparable to native PAF, MPAF failed to do so (Figure 5B,C), indicating that the neuritogenic activity of PAF requires enzymatic processing by PAFAH.

To further confirm the requirement, hippocampal neurons were treated with PAF in the presence of the PAFAH inhibitors, methyl arachidonyl fluorophosphonate (MAFP) (Chen et al. 2007) or P11^65^. Both inhibitors markedly reduced PAF‐induced neuritogenesis, yielding neurite lengths comparable to PBS‐treated controls (Figure 5B,C; PAF+MAFP and PAF+P11). Because PAFAH hydrolyses PAF to lyso‐PAF, we next assessed whether lyso‐PAF itself could reproduce this effect. Treatment of hippocampal neurons with lyso‐PAF failed to promote neuritogenesis, in contrast to PAF (Figure 5B,C). These findings indicate that the process of PAF metabolism by PAFAH, rather than the presence of PAF or its hydrolysis product lyso‐PAF, drives neurite formation. Together, these results identify neuronal PAFAH as the key mediator of PAF's neuritogenic function and provide mechanistic insight into its metabolism‐dependent mode of neuroregenerative action.

### 3.10 PAF Enhances Neuronal Cytoskeletal Remodeling via PAFAH‐Microtubule Protein Interactions

Building on these findings, we next investigated how PAFAH1B activity interfaces with the neuronal cytoskeleton. Given its known role in microtubule organization and neuronal migration (Arai et al. 2002), we examined whether PAF metabolism enhances interactions between PAFAH1B1 and cytoskeletal proteins to promote neurite stabilization and outgrowth. Immunoblot analysis revealed that PAF significantly increased levels of β3‐tubulin and high‐molecular‐weight MAP2 (h‐M) in NE‐4C neural stem cells within 48 h, while MPAF failed to induce comparable upregulation (Figure 5D). Notably, a 3‐day treatment with PAF did not drive NE‐4C cells toward neuronal differentiation, as these cells retained SOX2 and Nestin expression and did not form compact aggregates, morphological features observed during retinoic acid (RA)‐induced neuronal differentiation (Figure S5A,B). Therefore, the rapid increase in β3‐tubulin and MAP2 expression following PAF exposure likely reflects metabolic stimulation of cytoskeletal protein synthesis rather than differentiation‐induced gene expression.

To determine whether PAF facilitates direct interaction between PAFAH1B1 and microtubule‐associated proteins, we performed co‐immunoprecipitation assays in NE‐4C cells following PAF treatment. DCX and MAP2 are central regulators of microtubule stabilization and neurite extension (Korzhevskii et al. 2012). PAF enhanced the physical association between endogenous PAFAH1B1 and both DCX and low‐molecular‐weight MAP2 (l‐M) (Figure 5E,F). These interactions were absent in untreated control cells, suggesting that PAF specifically facilitates or stabilizes PAFAH1B1 binding to the neuronal cytoskeletal scaffold.

High‐ and low‐molecular‐weight MAP2 isoforms (h‐M and l‐M) reflect distinct neuronal maturation states: h‐M isoforms (MAP2A/B) are enriched in mature dendrites, while l‐M isoforms (MAP2C/D) are predominant in developing neurons and associated with neuronal plasticity and early neuritogenesis (Dehmelt et al. 2003). This pattern is consistent with our observation that PAF promotes neurite formation (Figures 1, 2‐ii and 3E). Together, these findings support a model in which PAF promotes neuronal structural maturation by facilitating PAFAH1B1‐microtubule protein interactions, thereby stabilizing MAP2 and β3‐tubulin expression and enhancing cytoskeletal organization. This PAFAH‐dependent mechanism provides new insight into how PAF mediates neural repair independently of classical receptor‐driven pathways.

### 3.11 PAF‐Enriched GWEVs Directly Promote Neuritogenesis in Developing Hippocampal Neurons

PAF is rapidly degraded in circulation by plasma PAFAH (PLA2G7) with a half‐life of only ∼30 s (Liu et al. 2011). Although the hydrolysis‐resistant analog MPAF exhibits a prolonged plasma half‐life (>100 min) (Kume and Shimizu 1997), it fails to exert therapeutic effects in models of brain injury (Figures 3 and 4). This lack of efficacy likely reflects MPAF's resistance to enzymatic degradation by PAFAH, which appears essential for PAF's regenerative action. Therefore, targeted delivery of unstable PAF to the brain is required to achieve therapeutic efficacy. Furthermore, because PTAFR‐mediated signalling is pro‐inflammatory (Upton et al. 2022), systemic PAF administration carries a risk of off‐target inflammation, particularly in PTAFR‐enriched tissues such as the spleen (Figure 4E). Stabilizing PAF within a protective carrier that enables brain‐specific delivery while minimizing peripheral exposure is thus critical for maximizing therapeutic benefit and minimizing systemic side effects.

To address this challenge, we evaluated whether PAF‐enriched GWEVs could act as an effective delivery platform to enhance neuritogenesis in hippocampal neurons. Although systemic GWEV administration improves cognitive function and neuronal regeneration (Chen et al. 2020, Chen et al. 2019), it remained unclear whether these effects arise from direct neuronal interaction or indirect systemic responses. To test for direct effects, we cultured primary hippocampal pyramidal neurons isolated at E17.5 with PBS, naïve EVs (EV), or GWEVs (Figure 5G). After 3 days, GWEV‐treated neurons exhibited significantly increased total neurite length compared with both PBS and naïve EV controls (Figure 5G,H), indicating a direct neuritogenic effect mediated by GWEVs. Moreover, GWEV‐treated neurons displayed enhanced neurite branching and network complexity, as confirmed by Sholl analysis (Figure 5G,I). GWEV‐induced neuritogenesis occurred in a dose‐dependent manner, with progressive increases in total neurite length across concentrations ranging from 1.7 × 10^7^ to 4 × 10^8^ EVs per well (Figures 5H and S6A). Importantly, this effect was consistently reproduced across multiple independent GWEV batches (Figure S6B,D), demonstrating robustness and reproducibility.

To further determine whether the GWEV‐mediated neuritogenesis depends on PAF cargo, primary hippocampal pyramidal neurons were cultured with GWEVs electroporated with either PAF‐neutralizing antibodies (GWEV‐αPAF) or control IgG (GWEV‐IgG) (Figure 5J,K) (Melo et al. 2014, Ishihara et al. 2000). After 3 days, GWEV‐IgG treatment significantly increased total neurite length, comparable to unmodified GWEVs (Figure 5J,K). In contrast, GWEV‐αPAF failed to promote neurite outgrowth, indicating that neutralization of PAF abolishes GWEV‐induced neuritogenesis (Figure 5J,K). Consistently, PAFAH inhibition also diminished the neuritogenesis‐promoting effect of GWEVs (Figure S6E,F), indicating that a significant portion of GWEV bioactivity is mediated through PAF metabolism.

Collectively, these findings demonstrate that PAF‐enriched GWEVs directly stimulate neuritogenesis in developing hippocampal pyramidal neurons, and that PAF metabolism acts as a critical effector of this response. Thus, GWEVs represent a stable, targeted and effective delivery system for PAF‐mediated enhancement of neuronal structural maturation and repair in the injured brain.

### 3.12 Development and Validation of a Bioorthogonal Labelling Strategy for Tracking GWEVs

To determine whether systemically administered PAF‐enriched GWEVs can directly reach damaged brain tissue, we developed an in vivo tracking strategy designed to preserve vesicle bioactivity. Given the limited accessibility of surface moieties on nanoscale EVs and the incomplete understanding of their biodistribution, we aimed to engineer a sensitive and minimally disruptive labelling method suitable for live‐animal imaging.

**FIGURE 6:**
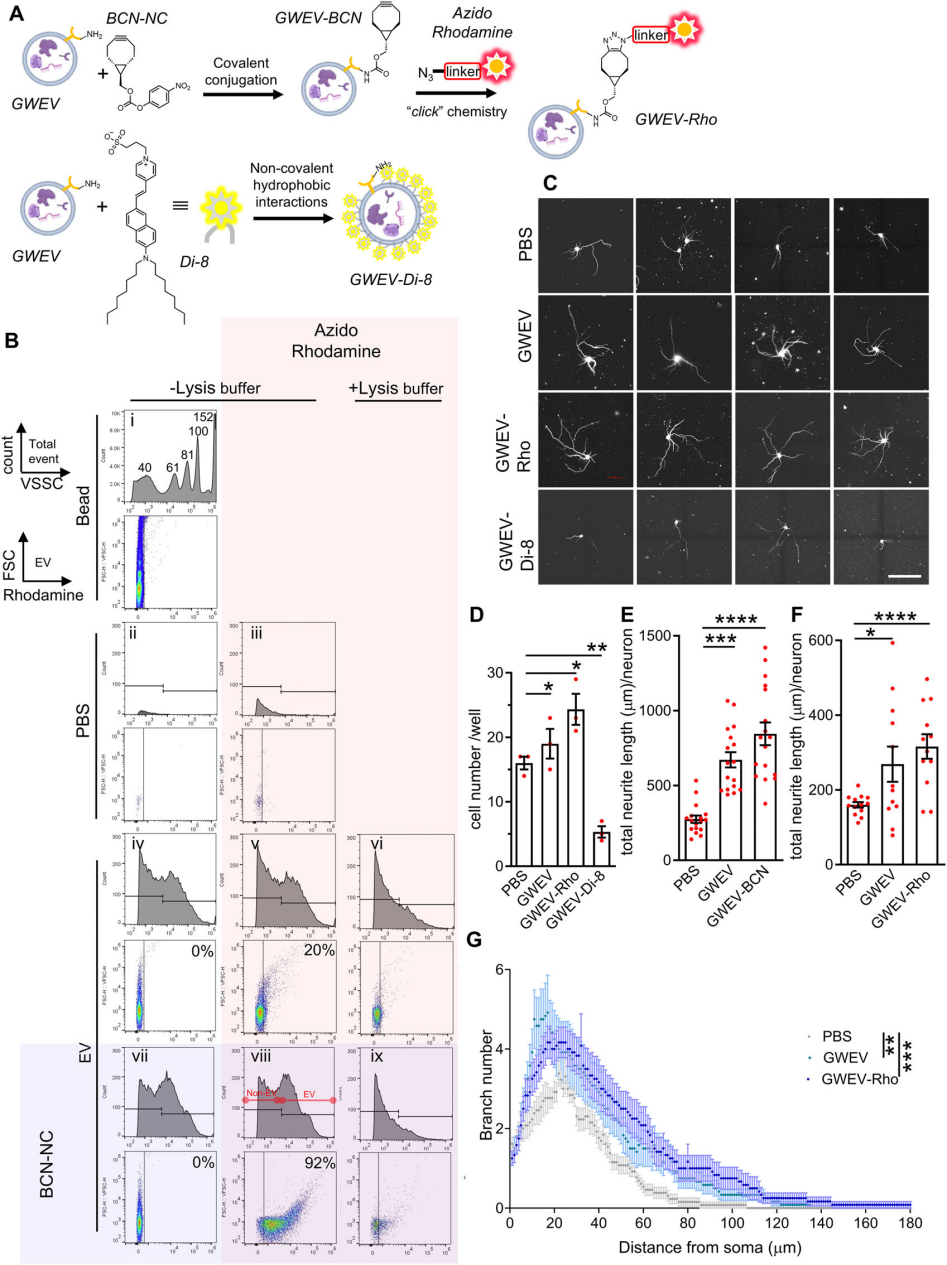
**SPAAC‐based bioorthogonal labelling enables efficient tracking of GWEVs while preserving their neuroregenerative function**. (A) Schematic overview of GWEV labelling strategies. Top: covalent surface labelling via SPAAC chemistry using BCN‐NC followed by azido‐rhodamine conjugation (GWEV‐Rho). Bottom: non‐covalent membrane labelling with the lipophilic dye Di‐8‐ANEPPS (GWEV‐Di‐8). (B) Nano‐flow cytometry analysis of labelling specificity. EVs were incubated with azido‐rhodamine alone (panel v), BCN‐NC alone (panel vii), or both sequentially (panel viii), and analysed for rhodamine fluorescence and particle size. Controls included PBS (panel ii), azido‐rhodamine alone (panel iii), unstained EVs (panel iv) and lysed EVs (panels vi, ix). Bead standards (panel i) served as size references. One‐colour histograms show log‐scale side scatter (VSSC) vs. particle count. Two‐colour dot plots show log‐scale Rhodamine intensity (from azido‐rhodamine) vs. log‐scale forward scatter (FSC). (C) Representative β3‐tubulin immunostaining of hippocampal neurons cultured for 3 days with PBS, unlabelled GWEVs, GWEV‐Rho, or GWEV‐Di‐8 (2 × 10^8^ vesicles/well). Scale bar: 100 µm. (D) Quantification of neuron counts per well in cultures described in (C). Data are mean ± SEM (*n* = 3). *p ≤ 0.05, **p ≤ 0.01. (E, F) Total neurite length in hippocampal neurons treated with GWEV‐BCN (E) or GWEV‐Rho (F) compared to PBS or GWEV controls. Data are mean ± SEM (E: *n* = 17, F: *n* = 12). *p ≤ 0.05, ***p ≤ 0.001, ****p ≤ 0.0001. (G) Sholl analysis of neurite arborization in neurons treated as in (F). Data are mean ± SEM (*n* = 12). **p ≤ 0.01, ***p ≤ 0.001.

We employed a covalent, bioorthogonal strain‐promoted azide‐alkyne cycloaddition (SPAAC) strategy. Figure S7A,B illustrate the synthetic reactions used to prepare the compounds for SPAAC‐based EV labelling; detailed procedures are provided in the Materials and Methods section. For covalent surface labelling, we conjugated bicyclo[6.1.0]nonyne nitrophenyl carbonate (BCN‐NC) to exposed lysine residues on the EV membrane, introducing a clickable alkynyl group (Figure 6A) (Toyokuni et al. 2003). The modified vesicles (GWEV‐BCN) were then reacted with azido‐rhodamine to generate fluorescently labelled GWEV‐Rho via SPAAC chemistry (Figure 6A). For comparison, we also labelled GWEVs non‐covalently using Di‐8‐ANEPPS, a membrane potential‐sensitive dye that associates with the EV membrane through amphipathic interactions, producing GWEV‐Di‐8 (Figure 6A). We then assessed labelling efficiency and bioactivity retention for both approaches.

Labelling efficiency was evaluated using nanoscale flow cytometry (CytoFLEX Nano), capable of resolving particles from 40 to 152 nm in diameter, calibrated using polystyrene beads detected via the Violet Side Scatter (VSSC) channel (Figure S8A, Bead). EV and non‐EV populations were distinguished based on histograms from PBS and serial EV dilutions (Figure S8A, EV and Non‐EV). These results demonstrated that the nano‐sized EV population could be clearly identified and separated from background noise using nanoscale flow cytometry, validating the system's capability for reliable EV detection and subsequent labelling efficiency analysis.

We next compared the labelling profiles of SPAAC‐based GWEV‐Rho and Di‐8‐labelled GWEVs. The EV population was identified via VSSC versus particle count histograms before and after lysis treatment (Figure S8A, green and blue curves, respectively). SPAAC‐based labelling selectively marked the EV population, while Di‐8 labelling showed nonspecific association with both EV and non‐EV particles (Figure S8A, red). To further quantify labelling specificity, EV samples were treated with PBS, BCN‐NC only, azido‐rhodamine only, or sequential BCN‐NC plus azido‐rhodamine, and analysed by nanoscale flow cytometry. The EV population was again identified by VSSC versus particle count distributions for PBS (Figure 6B‐ii, 6B‐iii) and EV samples with (Figure 6B‐vi, 6B‐ix) and without lysis buffer (Figure 6B‐iv, 6B‐v, 6B‐vii, 6B‐viii). The proportions of fluorescently labelled EVs were 0% for PBS and BCN‐NC alone, 20% for azido‐rhodamine alone and 92% for sequential SPAAC labelling, confirming both efficiency and specificity (Figure 6B). This shows that sequential click chemistry‐based labelling efficiently and selectively labelled the vast majority of GWEVs. Collectively, these results establish that the bioorthogonal surface labelling strategy using BCN‐NC and azido‐rhodamine provides high specificity and efficiency for EV labelling, enabling accurate in vivo tracking of PAF‐enriched GWEVs.

### 3.13 Bioorthogonal Labelling Preserves GWEV Bioactivity

In addition to evaluating labelling efficiency, we compared the effects of two labelling strategies on GWEV bioactivity: covalent SPAAC‐based labelling with BCN‐NC/azido‐rhodamine (GWEV‐Rho) and membrane labelling with the hydrophobic dye Di‐8‐ANEPPS (GWEV‐Di‐8). Primary hippocampal neurons isolated at E17.5 were cultured with PBS, unlabelled GWEVs, GWEV‐Rho and GWEV‐Di‐8 (Figure 6C). After 3 days, marked differences in neuronal viability were observed. GWEV‐Di‐8 treatment significantly reduced hippocampal neuron survival, indicating that membrane labelling with Di‐8 compromises EV function (Figure 6D). In contrast, GWEV‐Rho preserved the survival‐promoting effects of GWEVs, comparable to the activity of unmodified vesicles.

We next assessed whether bioorthogonally labelled GWEVs retained their ability to promote neuritogenesis. Quantitative analysis revealed that both GWEV‐BCN (Figure 6E) and GWEV‐Rho (Figure 6F) significantly increased total neurite length, mirroring the effect of unlabelled GWEVs. Moreover, Sholl analysis confirmed that GWEV‐Rho and native GWEVs similarly enhanced neurite branching and complexity, further indicating preservation of neurotrophic function (Figure 6G).

Collectively, these results demonstrate that bioorthogonal labelling using BCN‐NC/azido‐rhodamine preserves the regenerative bioactivity of GWEVs, including support for neuronal survival and neurite outgrowth. In contrast, membrane dye‐based labelling with Di‐8 markedly ablated GWEV function, justifying the use of click chemistry‐based labelling for tracking therapeutic EVs without compromising efficacy.

### 3.14 SPECT Imaging Reveals Injury‐Specific Brain Accumulation of GWEVs

**FIGURE 7:**
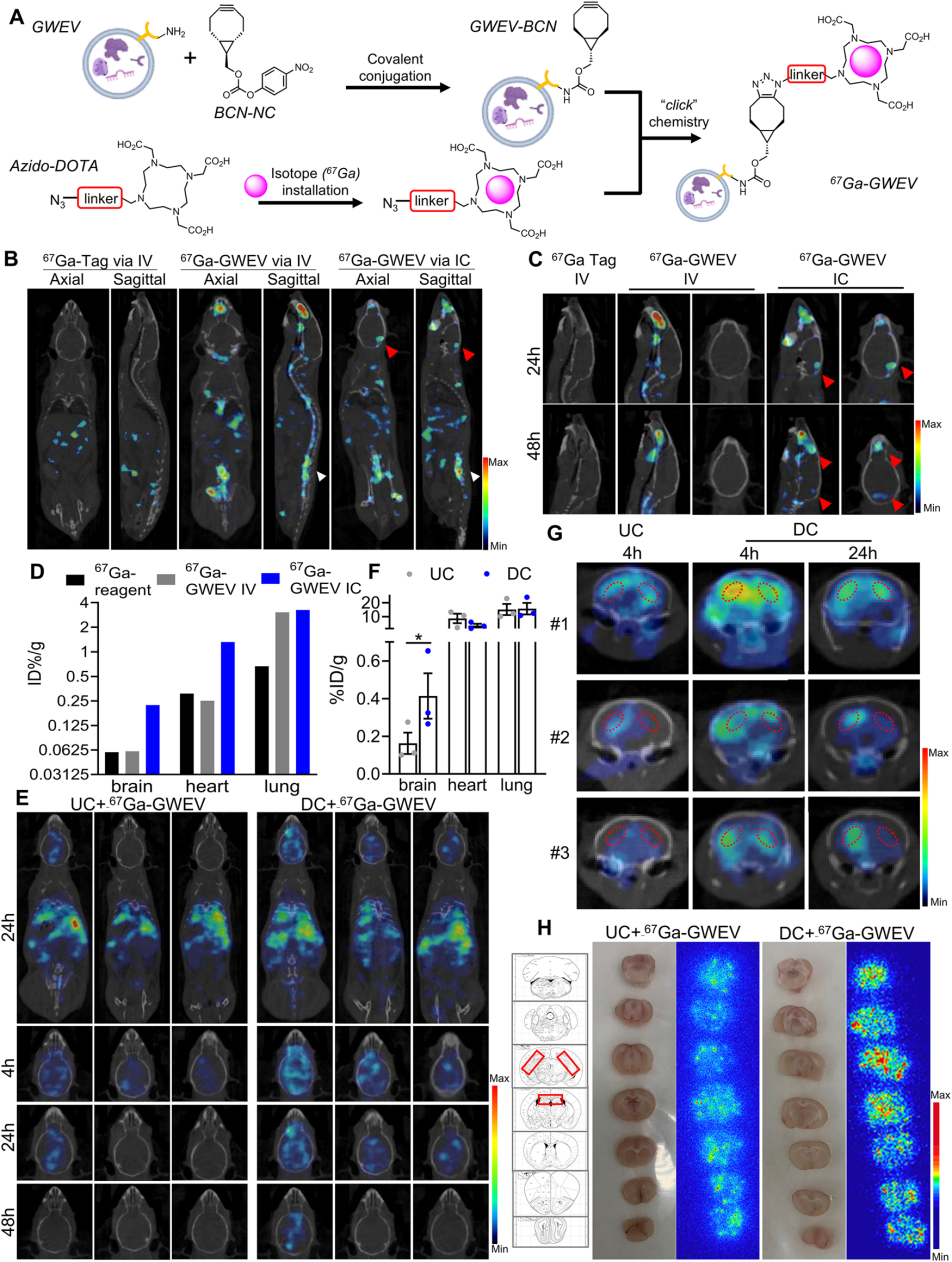
**Systemically administrated GWEVs preferentially accumulate in injured hippocampal regions**. (A) Schematic of bioorthogonal radiolabelling strategy for GWEVs. GWEVs were surface‐modified using BCN‐NC and conjugated with ^6^ ^7^Ga‐labelled azido‐DOTA via SPAAC chemistry. (B) Whole‐body SPECT images at 24 h post‐injection showing the in vivo biodistribution of ^6^ ^7^Ga‐GWEVs administered intravenously (IV) or intracardially (IC), compared to free ^6^ ^7^Ga‐DOTA (^6^ ^7^Ga‐Tag) in undamaged control (UC) mice (2.5 × 10^9^ vesicles/mouse). Images are shown at different body sections to illustrate overall biodistribution across tissues for each mouse. White arrowheads indicate intrathecal signal. Red arrowheads indicate radiotracer signal in the brain. (C) SPECT brain images of UC mice at 24 and 48 h post‐injection. Red arrowheads indicate radiotracer signal in the brain. (D) Ex vivo quantification of ^67^Ga‐GWEV distribution in major organs at 50 h post‐injection. Radioactivity is shown as the percentage of injected dose (%ID), normalized to organ wet weight (g). (E) *In vivo* SPECT images showing comparative biodistribution of IC‐injected ^6^ ^7^Ga‐GWEVs in UC and hippocampal injury (DC) mice at 4, 24 and 48 h post‐injection. Images are shown at different body sections to illustrate biodistribution across brain, liver, lung. (F) Ex vivo quantification of ^6^ ^7^Ga‐GWEV accumulation in brain and lung at 50 h post‐injection in UC and DC mice. Data are mean ± SEM (*n* = 3 mice per group). *p ≤ 0.05. (G) Brain SPECT images showing hippocampal localization of ^6^ ^7^Ga‐GWEVs at 4 and 24 h post‐injection in UC and DC mice. Sections encompassing the hippocampus were aligned across mice using a standardized brain atlas. Dashed outlines denote hippocampal regions, based on the mouse brain atlas. (H) Autoradiography of 1 mm‐thick coronal brain sections from UC and DC mice, 50 h post‐injection. Left panel shows anatomical reference with the hippocampal region boxed in red. Slice 3 exhibited the strongest radiotracer signal, corresponding to the injured hippocampus in DC mice. The colour scale represents relative SPECT signal intensity and is used for visualization purposes. Quantitative comparisons were derived from ex vivo radioactivity measurements, as shown in panels D and F.

To noninvasively track the biodistribution of GWEVs in vivo, we employed single‐photon emission computed tomography (SPECT), a nuclear imaging modality capable of detecting radiolabelled particles in living animals. Prior functional assays confirmed that GWEV‐Rho retains bioactivity following fluorescent labelling (Figure 6), supporting the adaptation of a similar bioorthogonal strategy for radiolabelling. We synthesized azido‐mono‐amide‐DOTA (azido‐DOTA; Figure S7C) and rapidly chelated it with the radioisotope ^6^ ^7^Ga. The resulting ^6^ ^7^Ga‐labelled DOTA was conjugated to GWEV‐BCN through a SPAAC reaction, yielding ^6^ ^7^Ga‐GWEVs, facilitating *in vivo* SPECT imaging (Figure 7A).

To examine the impact of delivery route on GWEV biodistribution, we administered ^67^Ga‐labelled GWEVs into undamaged (UC) mice through either intravenous (IV) or intracardiac (IC) injection. As a negative control, mice received IV injection of ^67^Ga‐DOTA (^67^Ga‐Tag) alone, which was rapidly cleared from circulation and produced minimal signal at 24 h post‐injection (Figure 7B). In contrast, ^67^Ga‐GWEVs remained detectable in vivo and localized prominently to intrathecal spaces following both IV and IC delivery (Figure 7B, white arrowheads), indicating central nervous system (CNS) access. The presence of ^67^Ga‐labelled GWEVs in cerebrospinal spaces suggests potential homing mechanisms, possibly mediated by the MSC origin of the EVs.

Notably, IC‐injected ^67^Ga‐GWEVs exhibited markedly higher brain accumulation than IV‐injected counterparts. At both 24 and 48 h post‐injection, SPECT imaging revealed higher radiotracer signals in the brain of IC‐injected mice (Figure 7B,C, red arrowheads). Quantitative ex vivo biodistribution analysis confirmed this difference: brain uptake was 3.7‐fold higher with IC delivery than with IV (0.22 vs. 0.06 %ID/g) (Figure 7D), while lung uptake remained comparable between the two routes (3.0 vs. 3.2 %ID/g).

Although the intrinsic spatial resolution of small‐animal SPECT imaging (∼0.8–1.0 mm voxel size) limits precise discrimination, we performed complementary confocal imaging to validate brain uptake. PKH26‐labelled GWEVs were administered via IC (3 × 10^9^ vesicles/mice) or IV (3 × 10^9^, 1 × 10^10^, or 2.6 × 10^10^ vesicles/mice), alongside dye‐only controls processed identically. Confocal imaging confirmed significantly higher brain PKH26 fluorescence in GWEV‐injected mice compared with controls (Figure S9). The brain signals increased with the IV dose of GWEVs in a dose‐dependent manner, indicating that the observed fluorescence reflects GWEVs that entered the brain parenchyma (Figure S9, bar chart). Importantly, brain fluorescence in mice receiving 3 × 10^9^ IC‐injected GWEVs was markedly higher than in those receiving 2.6 × 10^1^ ^0^ GWEVs intravenously, indicating greater brain accumulation through IC delivery (Figure S9, bar chart). Together, these results are consistent with the SPECT findings and demonstrate that GWEVs efficiently reach the brain, with IC administration achieving superior targeting. This delivery route substantially enhances GWEV brain tropism and may offer a strategic advantage for EV‐based therapies targeting central nervous system disorders, where efficient and selective delivery across the blood‐brain barrier is essential for therapeutic efficacy.

To assess whether GWEVs preferentially accumulate in injured brain tissue, we compared their biodistribution in UC mice and mice with hippocampal injury (DC), both receiving IC injections of ^67^Ga‐GWEVs. SPECT imaging revealed markedly higher brain signal intensity in DC mice than in UC mice at 4, 24 and 48 h post‐injection (Figure 7E). Notably, the brain signal persisted in all DC mice at 24 h, while it was rarely detected in UC mice at the same time point (Figure 7E, 24 h). Quantitative ex vivo analysis confirmed these findings: brain uptake of GWEVs was 2.6‐fold higher in DC mice than in UC mice (0.41 vs. 0.16 %ID/g) (Figure 7F), while lung uptake remained comparable between groups. The elevated brain accumulation of ^67^Ga‐GWEVs in DC mice enabled further analysis of the regional GWEV distribution across brain subregions using a mouse brain atlas. At 4 h post‐injection, ^67^Ga‐GWEVs selectively accumulated in hippocampus, the site of injury, in all DC mice, and the localization persisted up to 24 h. In contrast, hippocampal accumulation was not observed in all UC mice, even at the early time point (Figure 7G).

To further investigate the microdisposition of GWEVs in brain, we performed autoradiography on serial coronal brain sections spanning anterior to posterior regions (Figure 7H, left panel). These images revealed peak radiotracer intensity in slice 3, anatomically corresponding to the hippocampal region, in DC mice (Figure 7H). In UC mice (Figure 7C,H), no discrete accumulation was observed in the hippocampus, which is expected given the absence of brain injury. This injury‐specific accumulation demonstrates that GWEVs not only cross CNS barriers but also preferentially home to damaged brain regions.

**FIGURE 8:**
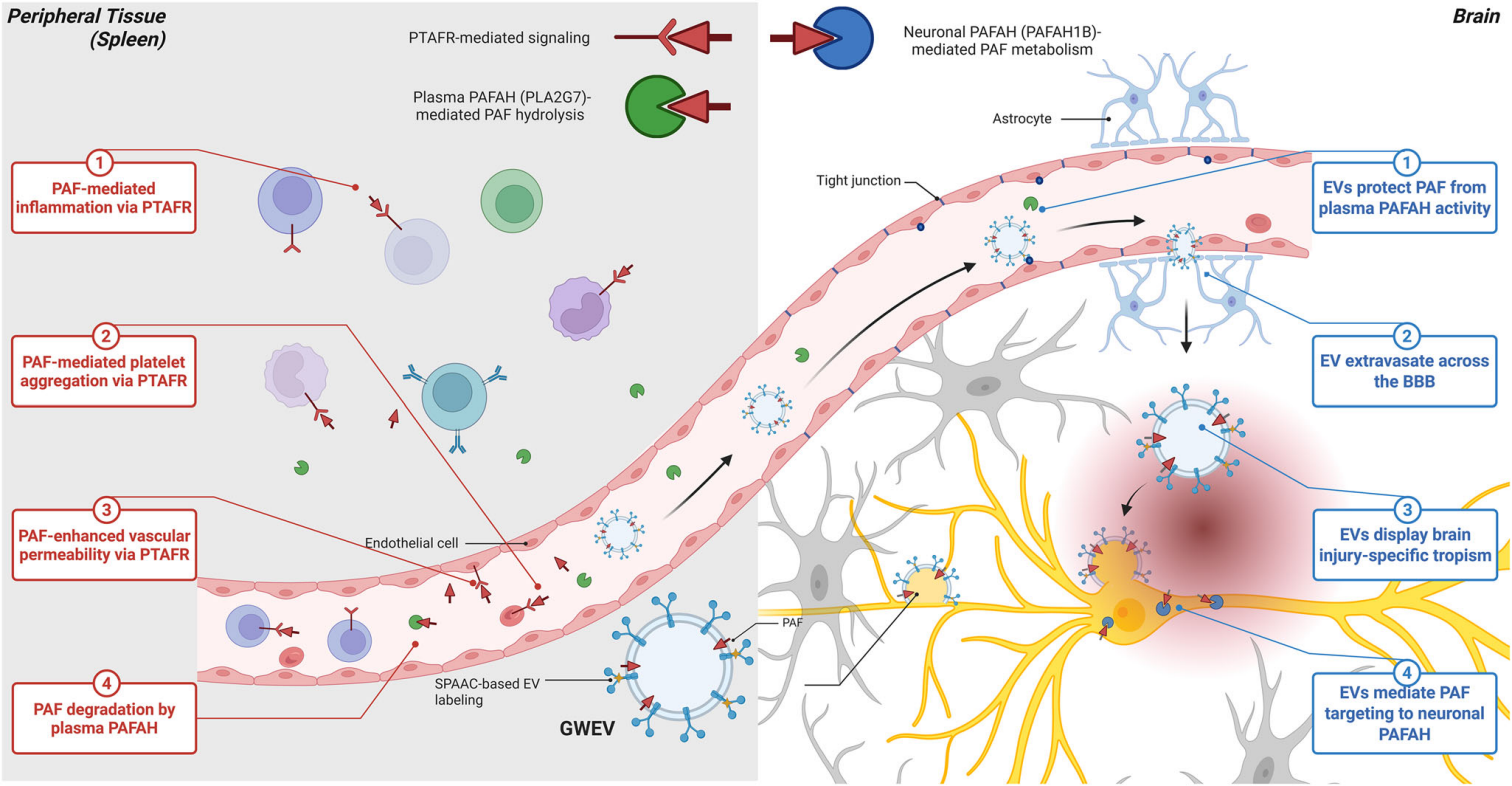
**Rationale for using GWEVs over free PAF in brain injury therapy**. Schematic illustration comparing the limitations of free PAF administration with the advantages of delivering PAF via engineered extracellular vesicles (GWEVs). Left panel (Peripheral Tissue): Systemically administered PAF acts on PTAFR‐expressing peripheral tissues such as the spleen, triggering proinflammatory and prothrombotic cascades, including (1) cytokine‐driven inflammation, (2) platelet aggregation, and (3) increased vascular permeability. Additionally, rapid degradation by plasma PAF‐acetylhydrolase (PAFAH/PLA2G7) limits bioavailability (4). Right panel (Brain): GWEVs encapsulate and protect PAF from enzymatic degradation (1), enable translocation across the blood‐brain barrier (2), and preferentially home to injured brain regions (3). Upon reaching the CNS, GWEVs deliver PAF directly to neurons, where it engages neuronal PAFAH to promote cytoskeletal remodelling and neurorepair (4). This delivery strategy enhances therapeutic efficacy while avoiding systemic inflammatory side effects, positioning GWEVs as a safer and more effective platform for targeted brain regeneration.

In conclusion, although PAF has demonstrated neuroregenerative potential, its clinical application is greatly hindered by systemic toxicity, off‐target inflammatory effects via PTAFR, and rapid degradation by plasma PAFAH. Figure 8 summarizes these challenges and illustrates the advantages of brain site‐delivery of PAF via PAF‐enriched extracellular vesicles (GWEVs) generated from MSCs under defined stimulation conditions. In this context, PAF may be delivered in a membrane‐associated form within the EV lipid bilayer (Liu et al. 2021, 2020), which protects it from enzymatic degradation in the circulation and enables efficient transport across the blood–brain barrier. Upon reaching the injured brain, GWEVs exhibit injury‐specific tropism and deliver PAF to neurons via EV–cell membrane interaction. Within neurons, PAF subsequently engages PAFAH‐dependent cytoskeletal remodelling pathways to promote neuronal repair, consistent with the established intracellular role of PAFAH1B1. This approach enhances therapeutic efficacy while minimizing systemic side effects, positioning GWEVs as a clinically viable alternative to free PAF for the treatment of brain injury. Furthermore, our data demonstrate that intracardiac administration provides efficient brain targeting and leads to selective enrichment of GWEVs in injured hippocampal tissue. Together, these findings support GWEVs as a promising biocompatible therapeutic ensemble for localized neurological repair.

## 4 Discussion

This study uncovers a metabolism‐driven mechanism of neuroregeneration in which the lipid mediator PAF, encapsulated within EVs, restores neuronal structure and cognitive function following hippocampal injury. The EVs used, termed GWEVs, are derived from EP4‐antagonist‐primed MSCs, which enhance both EV biogenesis and selective enrichment of metabolic cargo, particularly PAF. Systemic administration of GWEVs in a CA1 pyramidal neuron ablation model promoted neuronal regeneration, neuritogenesis, inflammation resolution and cognitive recovery, therapeutic effects not observed with naïve MSC EVs (Chen et al. 2019, 2020).

Although GW627368X has been reported to antagonize both the EP4 and thromboxane‐prostanoid (TP) receptors with comparable affinities (Wilson et al. 2006), our data and previous findings strongly suggest that the observed EV induction results specifically from EP4 blockade rather than TP receptor inhibition. In our earlier study developing the EV induction method in mammary stem cells (Lin et al. 2018), GW627368X robustly promoted EV release, whereas the selective TP receptor antagonist SQ29,548 did not elicit a similar effect. Moreover, inducible shRNA‐mediated knockdown of EP4 abolished GW627368X‐induced EV release, confirming that the response was EP4‐dependent (Lin et al. 2018). In line with reports that PGE_2_/EP4 signalling regulates MSC differentiation and stemness (Kulesza et al. 2023, Schmidt et al. 2024), we also observed that GW627368X modulated MSC phenotypes and lineage potential (Chen et al. 2019), further supporting a role for EP4 antagonism. Collectively, these findings indicate that the EV induction by GW627368X is mediated through selective disruption of PGE_2_/EP4 signalling rather than off‐target TP receptor blockade.

Through metabolomic profiling, PAF emerged as the most significantly enriched bioactive lipid in GWEVs. PAF is a well‐characterized phospholipid mediator, canonically acting through the G protein‐coupled receptor PTAFR to activate NF‐κB, MAPKs and proinflammatory cytokine production (Honda et al. 2002). However, transcriptomic analyses from the GTEx and Neuroscience Multi‐omics databases reveal that PTAFR is predominantly expressed in peripheral immune tissues, while PAFAH subunits, PAFAH1B1 (also known as LIS1), PAFAH1B2 and PAFAH1B3, are highly enriched in central nervous system (CNS) neurons (Figures 4E and 5A). This spatial segregation suggests a noncanonical, CNS‐specific mechanism whereby PAF acts through metabolic processing by neuronal PAFAH, rather than classical receptor signalling. Consistent with this hypothesis, MPAF, a PAFAH hydrolysis‐resistant PAF analogue with equivalent PTAFR affinity, fails to recapitulate the therapeutic effects of native PAF in injured hippocampus (Figure 4A–D). MPAF neither restores CA1 neuron numbers nor attenuates astrogliosis or microglial activation (Figure 3), highlighting the necessity of PAFAH‐mediated metabolism for PAF's neuroregenerative function. These findings establish a paradigm in which lipid bioactivity is shaped not solely by receptor engagement but critically by site‐specific enzymatic metabolism.

This mechanism aligns with the known roles of PAFAH1B1 in dynein‐mediated transport, microtubule stabilization and neurodevelopment (Arai et al. 2002). Mutations in PAFAH1B1 cause type I lissencephaly, characterized by impaired neuronal migration and cortical organization (Hirotsune et al. 1998). Here, we show that PAF enhances interactions between PAFAH1B1 and key cytoskeletal proteins such as MAP2 and DCX (Figure 5E,F), stabilizing microtubules and promoting neurite extension. In vivo, these effects translate to structural restoration of mature neurons (Figure 1H–J) and maturation of progenitor cells in the dentate gyrus (Figure 2), suggesting that PAF supports both architectural reinforcement and neurogenesis. PAF also reduced astrocyte and microglial activation following injury, effects not observed with MPAF, suggesting that its anti‐inflammatory benefits are mediated through metabolic processing rather than direct PTAFR signalling (Figure 3E,F). Given that PAFAH is enriched in neurons and PTAFR is predominantly expressed in microglia (Figure 5A), we propose that PAF metabolism by neuronal PAFAH facilitates neurorepair while limiting the release of inflammatory mediators, thereby reducing glial activation. These findings highlight a neuron‐centric, metabolism‐dependent model of neuroimmune modulation.

Although adult neurogenesis is largely confined to the dentate gyrus (DG) and subventricular zone (SVZ), our findings do not suggest that de novo neurogenesis occurs in the CA1 region of PAF‐treated mice within the short recovery period examined. Instead, the structural restoration observed in CA1 appears to result from neuritogenesis and glial modulation rather than the generation of new neurons (Figures 1 and 3). PAF treatment enhanced neurite extension in pyramidal neurons and reduced astrocytic reactivity, as evidenced by decreased GFAP expression, alongside the concurrent development of DCX‐positive progenitors within the DG. These results align with previous reports demonstrating that recovery of dendritic architecture and synaptic function can occur independently of neurogenesis, such as rapid restoration of dendritic structure via TrkB activation within minutes (Gao et al. 2025) and reversal of spine deficits following mGluR5 inhibition within 24 h (Li et al. 2025). Moreover, astrocytic modulation of neuroinflammation has been shown to enhance neuronal survival and network function (Chávez et al. 2019), supporting the interpretation that PAF promotes neuronal repair in CA1 by stabilizing existing neurons and restoring connectivity. Additionally, functional recovery in CA1 may be indirectly facilitated by PAF‐driven neurogenesis in the DG (Figure 2) through the DG‐CA3‐CA1 trisynaptic pathway (Stepan et al. 2015, Botterill et al. 2019, Mchugh et al. 2022). Thus, our data support a model in which PAF enhances CA1 integrity and cognitive recovery primarily through neurite regeneration and astrocyte regulation, complemented by DG‐mediated network remodelling, rather than by rapid local neurogenesis in CA1.

Despite its therapeutic potential, PAF's clinical application has been hampered by extreme instability in circulation and systemic inflammatory risks. Circulating PAF has a half‐life of approximately 3 min and is undetectable within 30 s of intravenous administration (Liu et al. 2011). This instability poses a substantial barrier to achieving therapeutic concentrations at target sites, especially within the central nervous system (CNS). Furthermore, PAF's therapeutic potential in the CNS is also limited by its inability to cross an intact blood‐brain barrier (BBB) under physiological conditions (Kumar et al. 1988). In addition to rapid degradation, PAF's systemic administration carries a narrow therapeutic window. Systemic PAF doses ≥10 µg/kg induce deleterious inflammatory effects, including vascular leakage and lethality (Young et al. 1985, Abhilasha et al. 2019, Jacob et al. 2016, Brambilla et al. 1987), whereas lower doses (1.0‐1.5 µg/kg) increase PAFAH activity without overt injury (Montrucchio et al. 2000, Wang et al. 1997, Sirois et al. 1994). Thus, effective therapeutic application of PAF requires both dose control and precise tissue targeting to avoid off‐target effects and systemic inflammation.

In the damaged control (DC) mice used in this study, BBB integrity is compromised, reflecting pathological changes that occur following brain injury. In our previous work (Chen et al. 2019), we observed disorganization of the tight junction protein claudin‐5 and altered association with astrocytic GFAP in the hippocampal CA1 region, consistent with vascular leakage and barrier disruption following injury. Such pathological permeability likely allows intracardially injected PAF to access the hippocampal parenchyma, where it can act locally to promote neuronal repair. In this study, intracardiac (IC) administration of PAF at 1.5 µg/kg (∼30 ng per 20 g mouse), given twice, effectively restored hippocampal structure without triggering systemic toxicity (Figures 1, 2, 3, 4). By contrast, intravenous (IV) delivery at the same dose failed to produce therapeutic effects (Figure 4F,G), likely due to rapid clearance and peripheral sequestration. These findings underscore the necessity of a targeted delivery vehicle, such as GWEVs, to enable effective brain delivery of PAF while minimizing systemic exposure.

To address these pharmacokinetic, safety and BBB‐targeting challenges, we developed GWEVs as a targeted delivery platform to stabilize PAF, protect it from degradation by plasma PAFAH, prevent off‐target PTAFR activation, and facilitate brain entry. Importantly, therapeutic efficacy was achieved with a low PAF dose (∼7.5 ng PAF per mouse) delivered via 15 µg GWEVs (Figure 1C) (Chen et al. 2020, Chen et al. 2019). To track biodistribution targeting and validate CNS delivery, we established a SPAAC‐based click‐labelling strategy compatible with SPECT and fluorescence imaging. Unlike conventional lipophilic dyes (e.g., DiR, Di‐8‐ANEPPS), which compromise EV integrity, our approach maintained GWEV bioactivity while enabling high‐resolution imaging (Figure 6). SPECT imaging confirmed preferential accumulation of radiolabelled GWEVs in injured hippocampus (Figure 7), likely reflecting the intrinsic homing properties of MSC‐derived vesicles and the increased permeability of damaged brain vasculature. This click‐labelling strategy offers a generalizable solution for tracking bioactive, lipid‐rich EVs in translational applications.

Together, these results position PAF‐enriched GWEVs as a scalable and metabolically rationalized platform for targeted brain repair. This study elucidates a previously underappreciated role for lipid metabolism in CNS repair and identifies PAFAH1B1 as a therapeutic node linking cytoskeletal remodelling with metabolic control. Our work has broader implications for the field of metabolic therapeutics, suggesting that restoring metabolic flux, not just receptor engagement, is critical for activating regenerative pathways in the brain. This study not only advances our understanding of lipid‐mediated neural repair but also introduces a translational strategy for CNS‐targeted regenerative therapeutics using engineered EVs.

## Author Contributions

**Shih‐Yin Chen**: methodology, software, data curation, investigation, validation, formal analysis, writing – original draft, writing – review and editing, conceptualization. **Jing‐Ya Hsu**: methodology, data curation, investigation. **Chen‐Fu Lo**: methodology, data curation, investigation, validation. **Yu‐Wei Liu**: methodology, data curation, investigation, validation. **Wei‐Neng Liao**: methodology, data curation, investigation, validation, formal analysis. **Wen‐Ting Luo**: methodology, software, data curation, investigation, validation, formal analysis, writing – review and editing, writing – original draft. **Yu‐Ju Chen**: methodology, investigation, validation. **Jui‐Ping Li**: methodology, investigation, validation, data curation, formal analysis. **Jen‐Kun Chen**: methodology, data curation, investigation, validation, formal analysis, supervision, writing – review and editing, conceptualization. **Lun Kelvin Tsou**: methodology, data curation, investigation, validation, formal analysis, supervision, writing – review and editing, conceptualization. **Hua‐Jung Li**: conceptualization, methodology, software, data curation, supervision, formal analysis, validation, investigation, funding acquisition, visualization, project administration, resources, writing – review and editing, writing – original draft.

## Conflicts of Interest

The authors declare no potential conflicts of interest.

## Supporting information



Supporting Information: jev270241‐sup‐0001‐figuresS1‐S9.pdf

Supporting Information: jev270241‐sup‐0002‐tableS1.xlsx

## Data Availability

The data that support the findings of this study are available from the corresponding author upon reasonable request.
