# Supplementary material for: Lipid Metabolism‐Driven CNS Repair via Targeted EV Delivery of PAF to Neurons
Source: J Extracell Vesicles. 2026 Feb 19;15(2):e70241. doi: 10.1002/jev2.70241 (PMC12919374; doi:10.1002/jev2.70241)
Supplement: Supplementary file 1 — Supporting Information: jev270241‐sup‐0001‐figuresS1‐S9.pdf [file JEV2-15-e70241-s002.pdf]

Lipid Metabolism-Driven CNS Repair via Targeted EV Delivery of PAF to Neurons

Supplementary Figures

Supplementary Figure 1

A

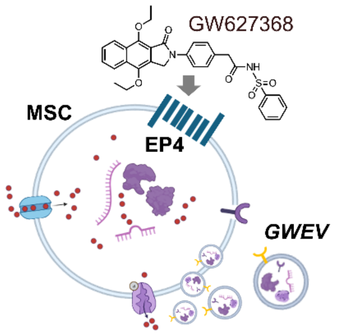

B

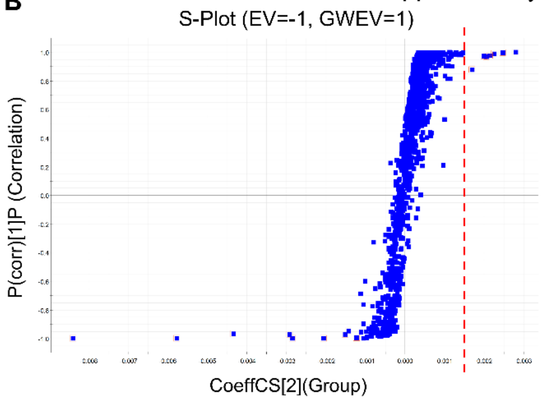

C

organic phase	positive mode			
		ID	Formula	Fold change (GWEV/EV)
	1	Platelet-activating factor C-16	C26H54NO7P	1st sample : 13.36<br>2nd sample : 11.43
		LysoPC(18:0)	C26H54NO7P	
	2	LysoPE(0:0/22:4(7Z,10Z,13Z,16Z))	C27H48NO7P	1st sample : 11.07<br>2nd sample : 1012/0
		LysoPE(22:4(7Z,10Z,13Z,16Z)/0:0)	C27H48NO7P	
	3	LysoPC(20:3(8Z,11Z,14Z))	C28H52NO7P	1st sample : 12.14<br>2nd sample : 10.14
		LysoPC(20:3(5Z,8Z,11Z))	C28H52NO7P	
	4	Hovenidulcigenin B	C32H50O7	1st sample : 18.5<br>2nd sample : 1219/0
		3-O-α-L-mycarosylerythronolide B	C28H50O10	
	negative mode			
		ID	Formula	Fold change (GWEV/EV)
	1	LysoPC(17:0)	C25H52NO7P	1st sample : 6.5<br>2nd sample : 13.36
		LysoPE(0:0/20:0)	C25H52NO7P	
		LysoPE(20:0/0:0)	C25H52NO7P	
	2	PC(15:0/22:4(7Z,10Z,13Z,16Z))	C45H82NO8P	1st sample : 2.93<br>2nd sample : 713/0
		PC(22:4(7Z,10Z,13Z,16Z)/15:0)	C45H82NO8P	
		PE(18:0/22:4(7Z,10Z,13Z,16Z))	C45H82NO8P	
		PE(18:2(9Z,12Z)/22:2(13Z,16Z))	C45H82NO8P	
		PE(18:3(6Z,9Z,12Z)/22:1(13Z))	C45H82NO8P	
		PE(18:3(9Z,12Z,15Z)/22:1(13Z))	C45H82NO8P	
		PE(18:4(6Z,9Z,12Z,15Z)/22:0)	C45H82NO8P	
		PE(20:0/20:4(5Z,8Z,11Z,14Z))	C45H82NO8P	
		PE(20:0/20:4(8Z,11Z,14Z,17Z))	C45H82NO8P	
		PE(20:1(11Z)/20:3(5Z,8Z,11Z))	C45H82NO8P	
		PE(20:1(11Z)/20:3(8Z,11Z,14Z))	C45H82NO8P	
aqueous phase	negative mode	ID	Formula	Fold change (GWEV/EV)
	1	Estriol 3-sulfate 16-glucuronide	C24H32O12S	1st sample : 42945/0<br>2nd sample : 6521/0
		1,5-Diferuloylquinic acid	C27H28O12	
		Methyl 3,4-dicaffeoylquinate	C27H28O12	
	positive mode			

Supplementary Figure 1. GWEVs contain elevated levels of platelet-activating factor (PAF). (A) Schematic overview illustrating the induction of extracellular vesicle (EV) release by the EP4 antagonist GW627368X, generating GWEVs from MSCs. (B) S-plot derived from mass spectrometry

(MS) analysis highlighting metabolomic differences between EVs and GWEVs. Red dashed line indicates the threshold for metabolite enrichment in GWEVs. (C) List of putative metabolites enriched in GWEVs, identified under both positive and negative electrospray ionization (ESI) modes.

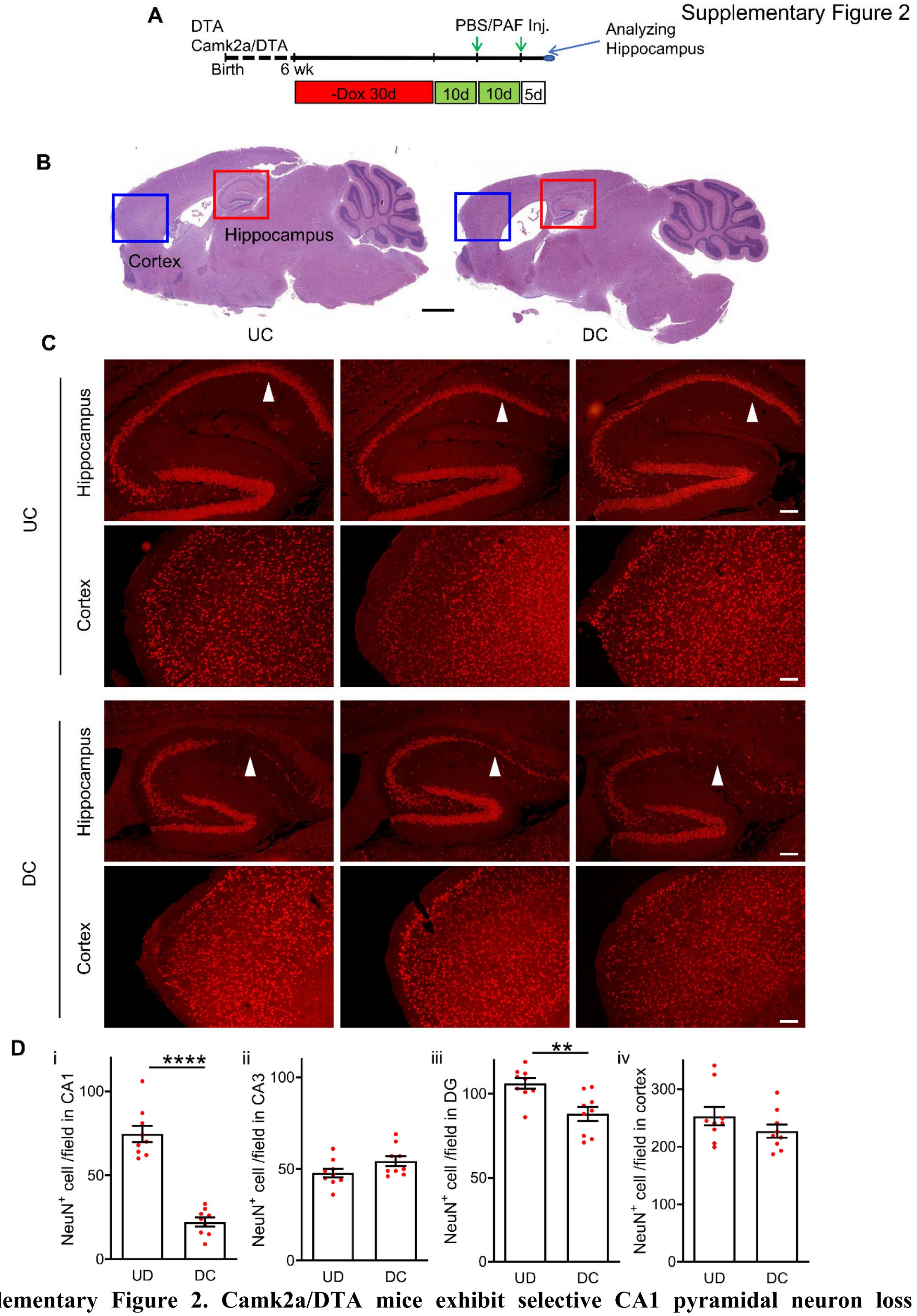

Supplementary Figure 2. Camk2a/DTA mice exhibit selective CA1 pyramidal neuron loss following Dox withdrawal, confirming effective lesioning. (A) Experimental timeline illustrating induction of hippocampal damage, intracardiac PAF administration (1.5 μg/kg), and subsequent tissue collection. (B) Hematoxylin and eosin staining of the hippocampus in Dox-withdrawn tetO-DTA mice (DTA, undamaged control [UC]) and Camk2a-tTA/tetO-DTA mice (Camk2a/DTA, damaged control [DC]). Scale bar: 1000 μm. The blue box indicates the cortical region analyzed by staining, and the red box indicates the hippocampal region analyzed. (C) Immunofluorescence staining of NeuN in forebrain

including the cortex and hippocampus, from UC, DC, and DC+PAF mice at 5 days post-treatment. Scale bar: 100 µm. (D) Quantification of NeuN-positive cells corresponding to the regions shown in panel C. Panel i shows the CA1 region, panel ii shows the CA3, panel iii shows the DG, and panel iv shows the cortex. Data are mean ± SEM (n = 9 fields per group). **P ≤ 0.01, ****P ≤ 0.0001. Field area for hippocampal regions: 10,000 μm^2; field area for cortical regions: 100,000 μm^2.

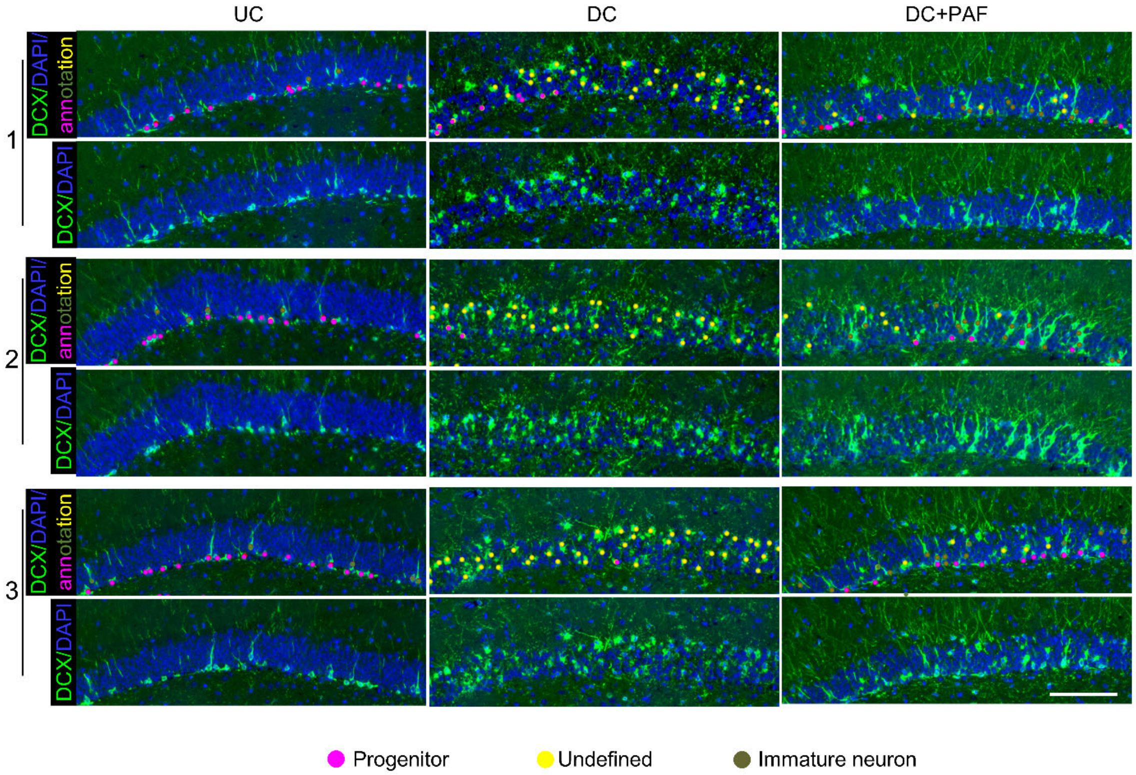

Supplementary Figure 3. Immunofluorescence staining of DCX in the dentate gyrus (DG). Immunofluorescence staining of DCX in the DG of UC, PBS-treated damaged (DC), and PAF-treated damaged (DC+PAF) mice five days post-treatment. Nuclei were stained with DAPI. DCX-positive cell types were annotated based on cell morphology and spatial position. Scale bar: 100 µm.

Supplementary Figure 4

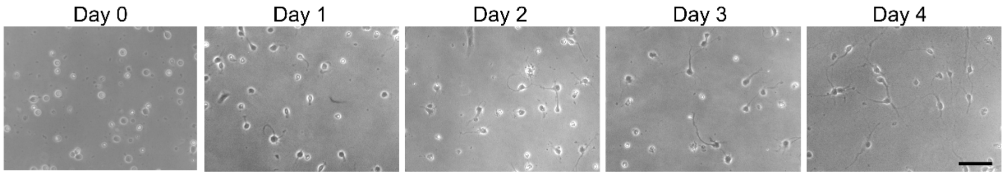

Supplementary Figure 4. The developmental progression of hippocampal neurons in vitro is characterized by morphological stages. Phase-contrast images showing morphological changes from day 0 to day 4 in primary hippocampal pyramidal neurons isolated at embryonic day 17.5. Scale bar: 100 μm.

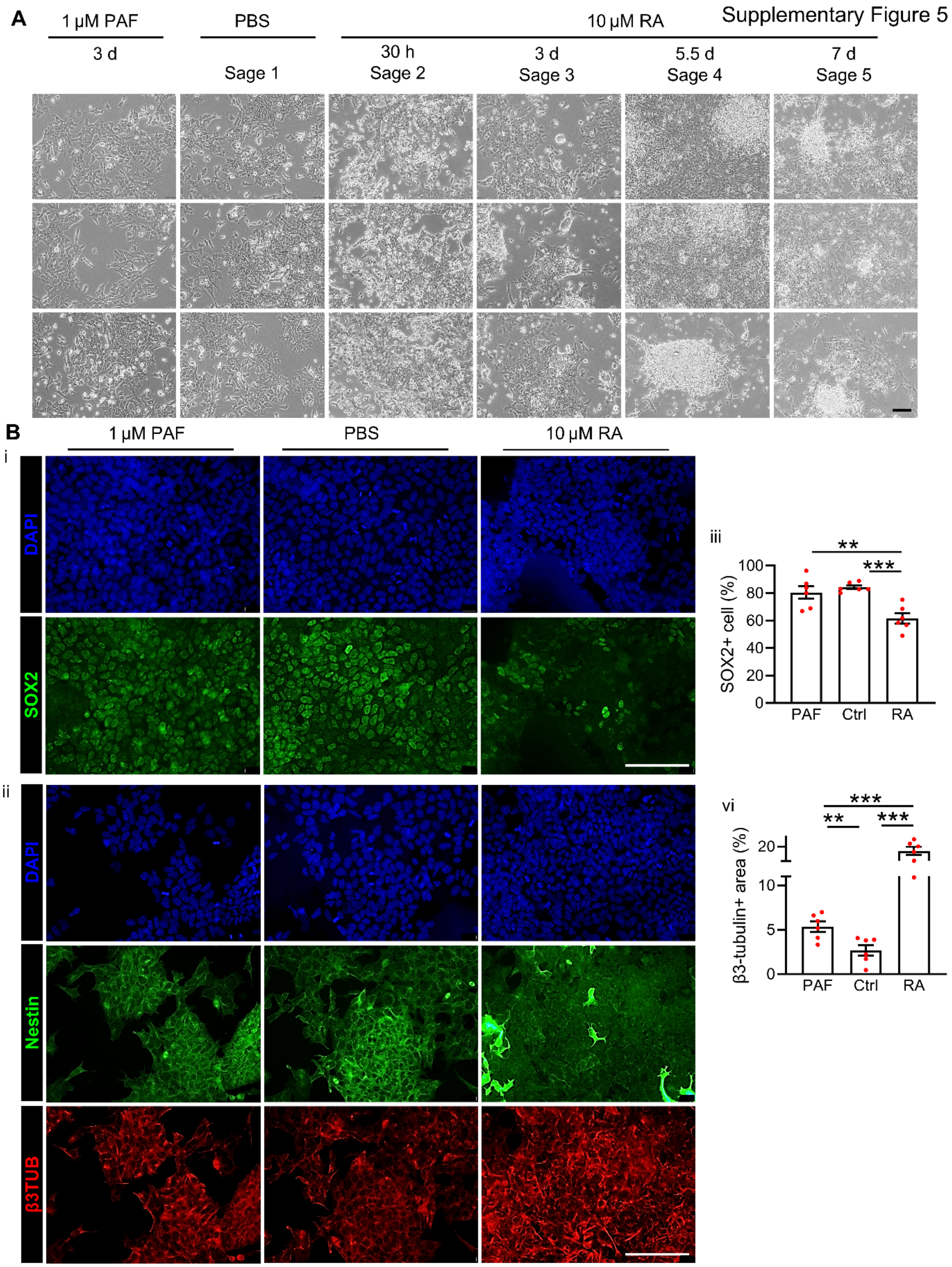

Supplementary Figure 5. PAF increases cytoskeletal protein expression in NE-4C neural stem cells without inducing neuronal differentiation. (A) Phase-contrast images showing morphological changes in NE-4C cells after treatment with retinoic acid (RA, 10 μM) or PAF (1 μM). Scale bar: 100 μm. "d" indicates days of treatment. (B) Immunofluorescence staining of SOX2 (panel i), Nestin, and β3-tubulin (panel ii) in NE-4C cells after 3-day treatment with PBS, RA (10 μM), or PAF (1 μM). Nuclei were counterstained with DAPI. Scale bar: 100 μm. Quantification of SOX2-positive cells and

β3-tubulin expression is shown in panel iii and iv, respectively. Data are mean ± SEM (n = 6 fields per group). $^{**}P \leq 0.01$, $^{***}P \leq 0.001$. Field area: 25,000 μm^2.

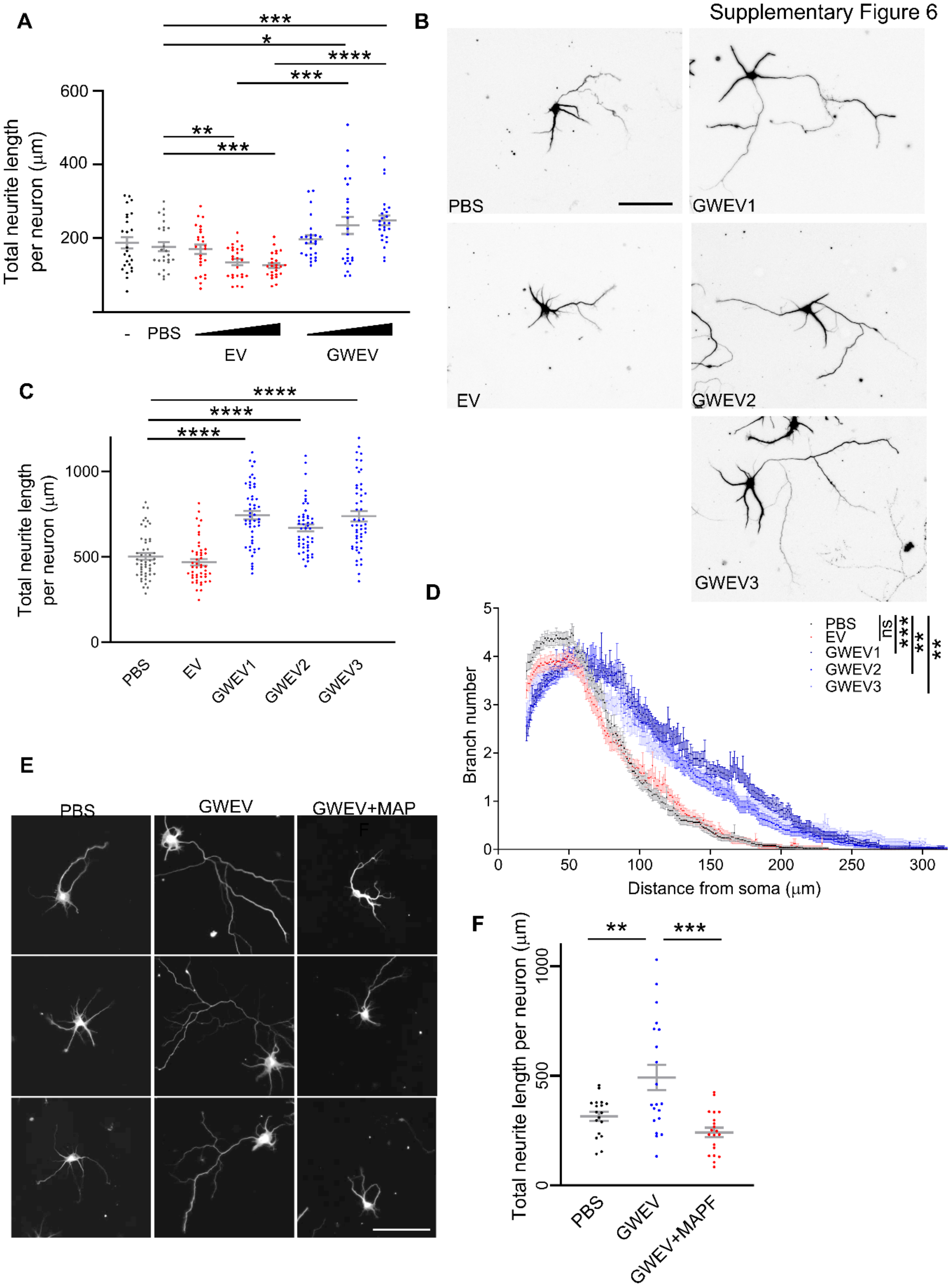

Supplementary Figure 6. Therapeutic efficacy of different batches of GWEVs on hippocampal neurons. (A) Quantification of neurite length of hippocampal neurons cultured with PBS, EVs (1.7×10^7, 3.3×10^7, 6.7×10^7 vesicle/well), or increasing doses of GWEVs (1.7×10^7, 3.3×10^7, 6.7×10^7 vesicle/well) at day 3 after isolation. Data are mean ± SEM (n = 25). *P ≤ 0.05, **P ≤ 0.01, ***P ≤ 0.001, ****P ≤ 0.0001. (B) Immunofluorescence staining of β3-tubulin in hippocampal neurons cultured with PBS, EVs (4×10^8 vesicle/well), or three different batches of GWEVs (4×10^8/well) at day 3 after isolation. Scale bar: 100 μm. (C) Quantification of neurite length of hippocampal neurons

described in panel B. Data are mean ± SEM (n = 50). ****$P \leq 0.0001$. (D) Sholl analysis of neurite intersections in neurons from panel B. Data are presented as curves showing means ± SEM (n = 50). **$P \leq 0.01$, ***$P \leq 0.001$. (E) Representative β3-tubulin immunostaining of primary hippocampal neurons cultured for 3 days with PBS, GWEVs (1×10^8 vesicles/well), or GWEVs + MAFP (500 nM). Scale bar: 100 μm. (F) Quantification of total neurite length in hippocampal neurons described in (E). Data are mean ± SEM (n = 20 neurons per group). **$P \leq 0.01$, ***$P \leq 0.001$.

Supplementary Figure 7

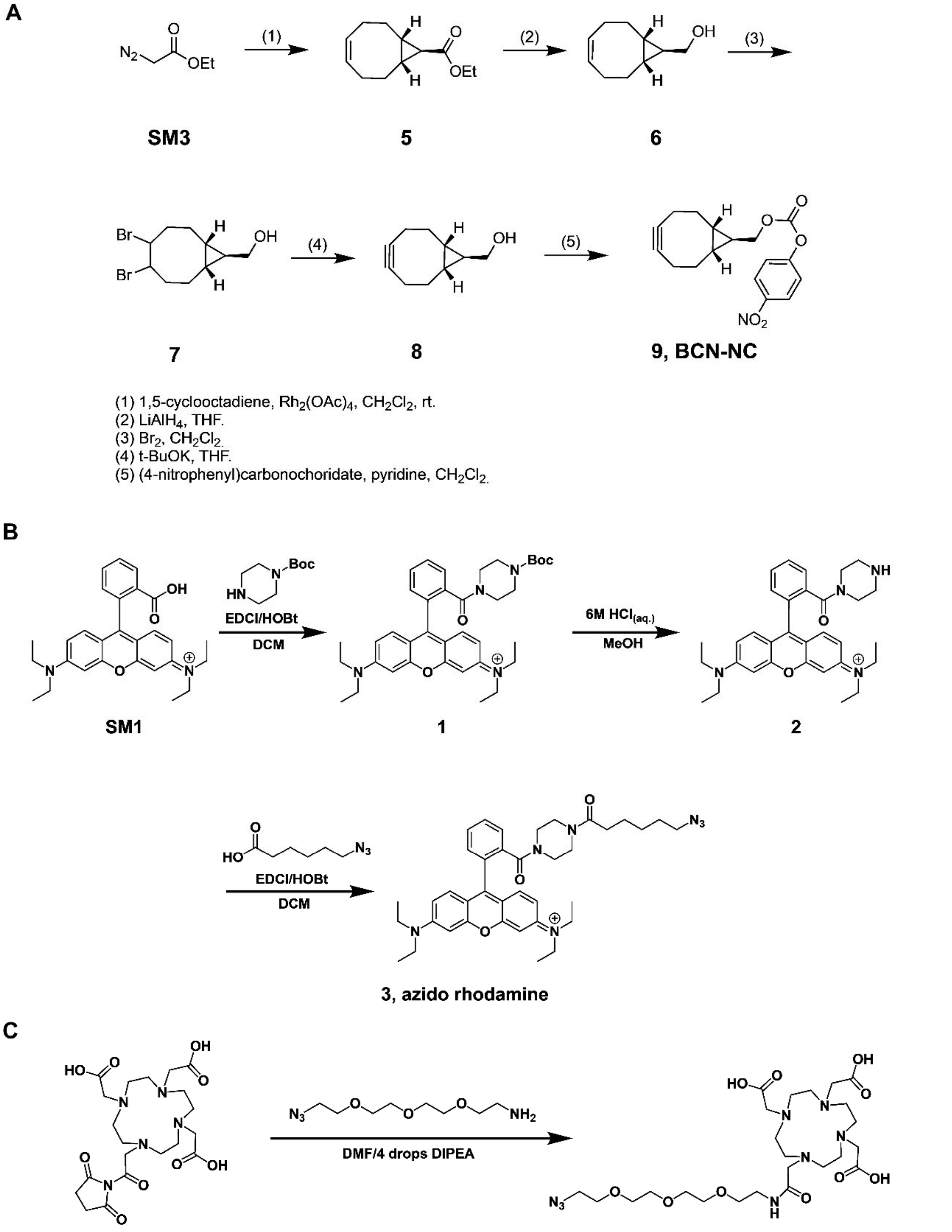

Supplementary Figure 7. Synthesis of compounds used for SPAAC-based EV labeling. (A) Synthesis of ((1R,8S,9r)-bicyclo[6.1.0]non-4-yn-9-yl)methyl (4-nitrophenyl) carbonate, (BCN-NC). (B) Synthesis of azido rhodamine. (C) Synthesis of azido-DOTA.

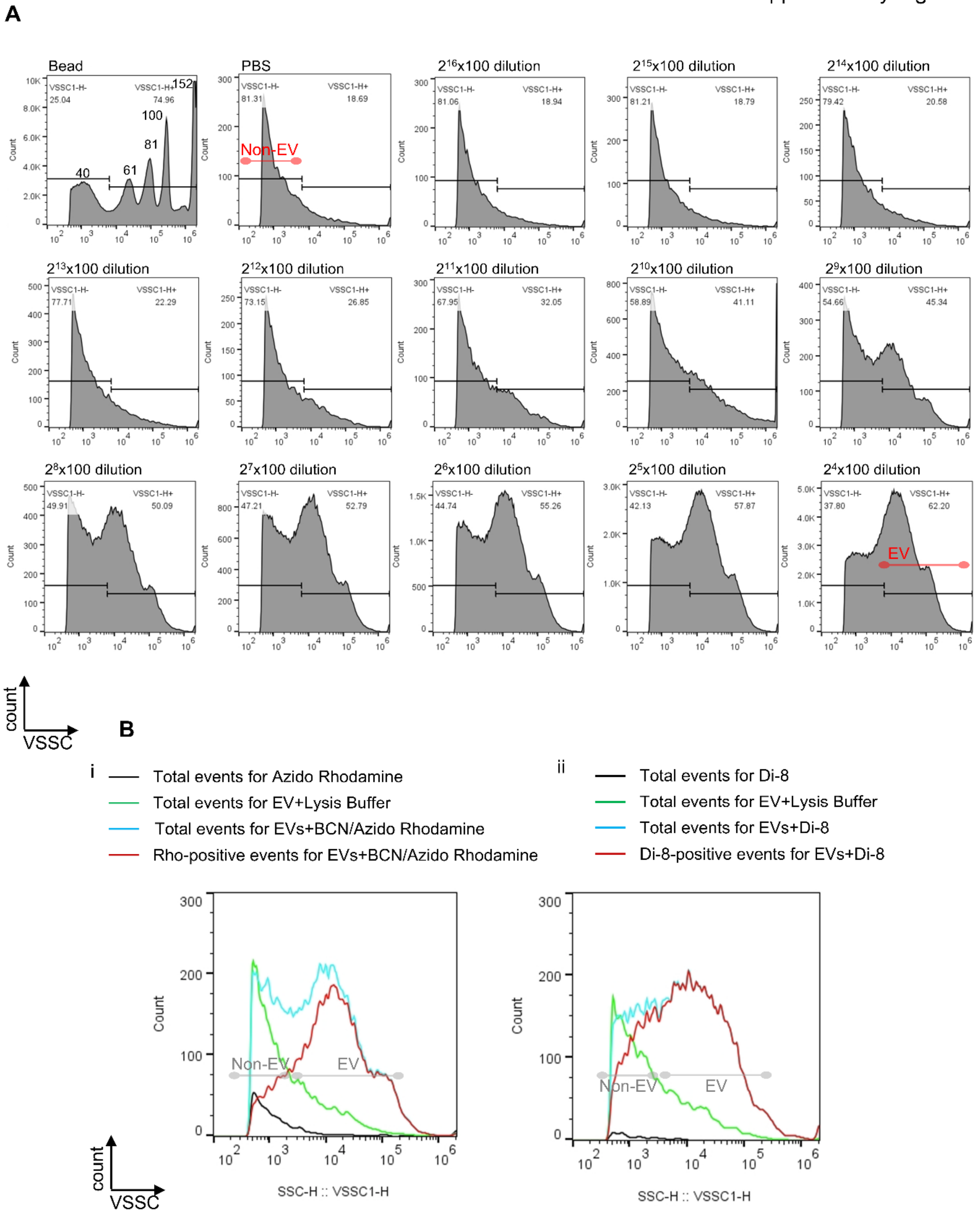

Supplementary Figure 8. A SPAAC-based strategy selectively labels the EV population. (A) Nano-flow cytometric analysis of serial dilutions of EVs. Histograms display log-scale Violet Side Scatter (VSSC) vs. particle count. PBS was used as a diluent control. Bead standards were used as size references. (B) Nano-flow cytometric analysis of EV labeling using either BCN/azido-rhodamine (panel i) or Di-8-ANEPPS (panel ii), assessed with a CytoFLEX nano-flow cytometer. Histograms show log-scale VSSC versus particle count. Black, total events for dye-only control; green, total events for EVs treated with lysis buffer (EV negative control); blue, total events for EV with dye, Red: dye-positive events for EV with dye.

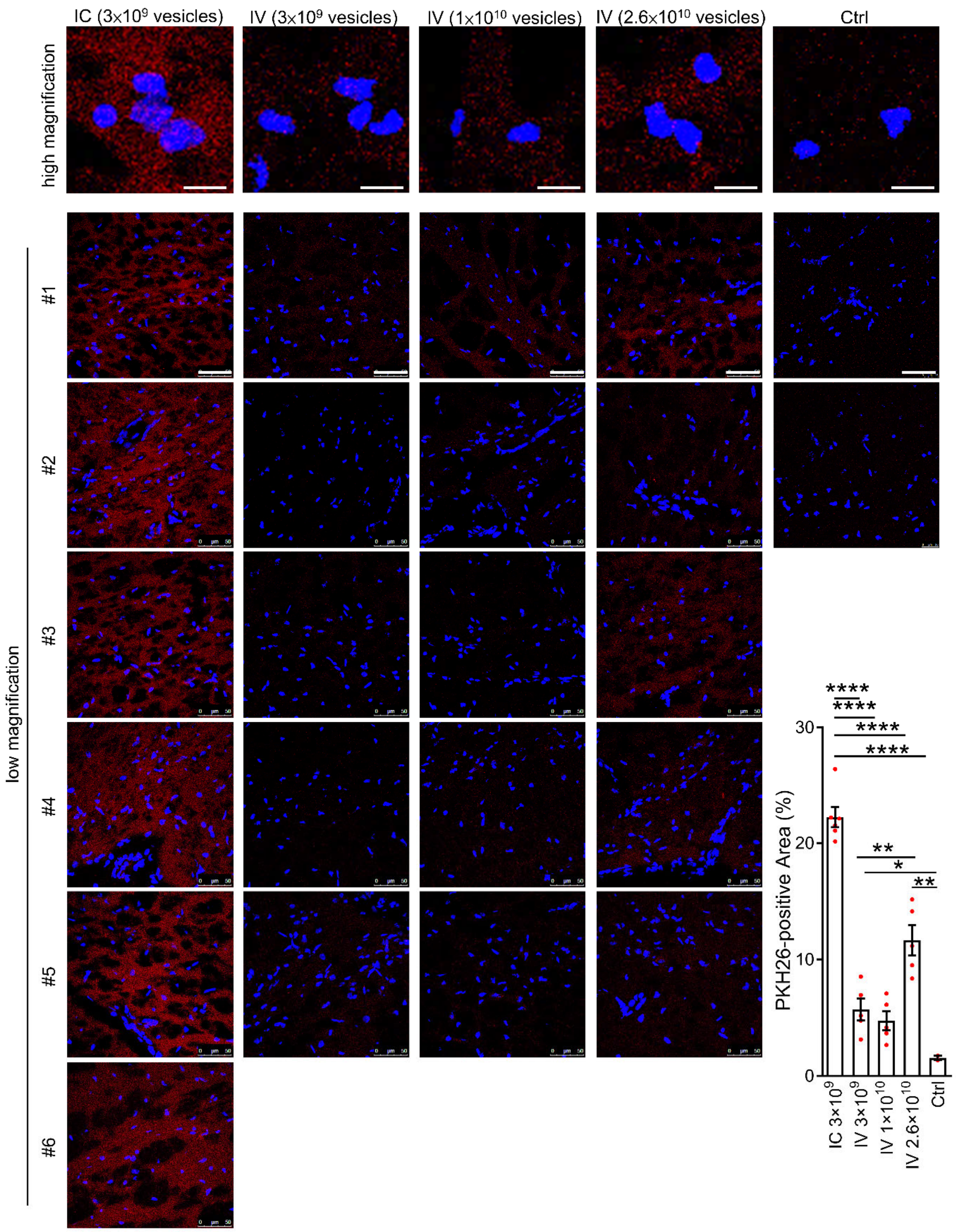

Supplementary Figure 9. GWEV uptake in the mouse brain. PKH26-labeled GWEVs and negative control samples (Ctrl; see Methods) were administrated into mice via intracardiac (IC) or intravenous (IV) injection. The number of vesicles injected per mouse is indicated in the figure. Sixteen hours post-injection, mice were sacrificed, and brains were collected for confocal imaging analysis. Cell nuclei were stained with DAPI (blue). Scale bar: 10 μm (high magnification) and 50 μm (low magnification). Quantification of PKH26-positive area in mice brain is shown in the accompanying bar chart. Data are means ± SEM (n = 5-6 mice per group). *P ≤ 0.05, **P ≤ 0.01, ****P ≤ 0.0001.
